# Association of dietary fatty acids with longitudinal change in plasma-based biomarkers of Alzheimer’s disease

**DOI:** 10.1016/j.tjpad.2025.100117

**Published:** 2025-03-18

**Authors:** Serena S. Hoost, Lawrence S. Honig, Min Suk Kang, Aanya Bahl, Annie J. Lee, Danurys Sanchez, Dolly Reyes-Dumeyer, Rafael A. Lantigua, Jeffrey L. Dage, Adam M. Brickman, Jennifer J. Manly, Richard Mayeux, Yian Gu

**Affiliations:** a Taub Institute for Research on Alzheimer’s Disease and the Aging Brain, Vagelos College of Physicians and Surgeons, Columbia University, 630 West 168th Street, New York, NY, 10032, USA; b G.H. Sergievsky Center, Vagelos College of Physicians and Surgeons, Columbia University, 630 West 168th Street, New York, New York, 10032, USA; c Department of Neurology, Vagelos College of Physicians and Surgeons, Columbia University, and the New York Presbyterian Hospital, 710 West 168th Street, New York, New York, 10032, USA; d Department of Epidemiology, Mailman School of Public Health, Columbia University, 722 West 168th Street, New York, New York, 10032, USA; e Department of Medicine, Vagelos College of Physicians and Surgeons, Columbia University, and the New York Presbyterian Hospital, 630 West 168th Street, New York, New York, 10032, USA; f Stark Neurosciences Research Institute, Suite 414, Indiana University School of Medicine, 320 West 15th Street, Indianapolis, Indiana, 46202, USA

**Keywords:** Blood-based biomarker, Diet, Omega-3, Alzheimer’s disease, Amyloid

## Abstract

**Background:**

Elevated intake of omega-3 polyunsaturated fatty acids is linked to a reduced risk of dementia in some prospective studies. However, few studies have examined the relationship between nutrient intake and plasma biomarkers of Alzheimer’s disease.

**Objectives:**

We explored whether omega-3, omega-6, and monounsaturated fat intakes were associated with changes in plasma biomarkers of Alzheimer’s disease over time.

**Design:**

The Washington Heights-Inwood Columbia Aging Project is a prospective cohort study (1994–2021); the data set used here includes a mean follow-up of 7.0 years.

**Setting:**

Community-based in New York City.

**Participants:**

599 dementia-free individuals at baseline who completed a 61-item food frequency questionnaire and had biomarkers measured in plasma from at least two different time points.

**Measurements:**

Fatty acid intake tertiles were computed from participant-completed 61-item Willett semi-quantitative food frequency questionnaires (Channing Laboratory, Cambridge, Massachusetts) obtained once at their baseline visit. Plasma-based biomarker assays were performed, using the single molecule array technology Quanterix Simoa HD-X platform, at baseline and follow-up visits. Generalized Estimating Equations (GEE) models were used to evaluate the association between baseline nutrient intake tertile and changes in biomarkers including phospho-tau181, amyloid-beta 42/40 ratio, phospho-tau181/amyloid-beta42 ratio, glial fibrillary acidic protein, neurofilament light chain, and two biomarker patterns derived from Principal Component Analysis (PCA1 and PCA2), with higher scores indicating a high level of neurodegeneration and low level of Alzheimer’s disease burden, respectively). Models were adjusted for age, sex, race/ethnicity, education, and calculated total energy intake initially, and additionally for cerebrovascular risk factors.

**Results:**

Higher baseline omega-3 intake tertile was associated with lesser decline in PCA2 (*β* = 0.221, *p* < 0.001) and amyloid-beta 42/40 ratio (*β* = 0.022, *p* = 0.003), and a lesser rise in phospho-tau181 (*β* = −0.037, *p* = 0.001) Higher omega-6 intake tertile was linked to a lesser rise in phospho-tau181 (*β* = −0.050, *p* < 0.001) and glia fibrillary acidic protein (*β* = −0.028, *p* = 0.002). Most associations persisted after adjusting for cardiovascular risk factors.

**Conclusions:**

Higher relative baseline intake of omega-3 and omega-6 fatty acids is associated with lesser progression of blood-based biomarkers of Alzheimer’s disease. Consuming healthy fatty acids may help prevent accumulation of Alzheimer’s disease-related pathological changes.

## 1. Introduction

Accumulating evidence suggests the importance of dietary factors in preventing Alzheimer’s disease (AD) [1]. Polyunsaturated fatty acids (PUFAs) stand out for their critical roles in brain structure and function and their consistent association with reduced risk of AD in epidemiological studies [1–3]. Individuals with higher dietary intake of omega-3 (O-3) PUFAs, found most richly in fish and seafood [4], are less likely to develop cognitive decline and AD in observational studies [5–8], including ours [7]. These associations raise the question of whether these key nutrients slow the progression of AD pathophysiology in the pre-clinical phase, when individuals may derive the greatest benefit from targeted lifestyle modifications. In AD, changes in the brain reflected in body fluid biomarkers, or increased amyloid burden on positron emission tomography (PET), may precede cognitive impairment by as many as 15 years [9–11].

Blood-based biomarkers provide an accessible way to identify individuals at risk of developing AD in large cohorts [12], and they allow for serial measurements tracking the accumulation of AD pathology over time [13]. As such, blood-based biomarkers may provide insight into the relationship between diet and the progression of AD pathophysiology before symptom onset. However, the association between dietary fatty acid intake and AD biomarkers, especially blood-based biomarkers, has been evaluated in few studies so far [14–19].

We chose to evaluate whether high intake of key nutrients from food would predict a lesser change in plasma AD biomarkers over time in participants of a multi-ethnic cohort of older adults who were dementia-free at baseline. We included dietary intake of O-3 and omega-6 (O-6) PUFAs and monounsaturated fatty acids (MUFAs) measured at baseline. We included phosphorylated tau 181 (P-tau181), amyloid beta (A*β*) 42/40 ratio, P-tau181/A*β*42 ratio, neurofilament light chain (NfL) and glial fibrillary acidic protein (GFAP) as AD-related biomarkers. We also included the first two biomarker patterns previously derived from principal component analysis (PCA1, PCA2) of the latter four biomarkers [20]. The PCA1 pattern has elevated NfL and GFAP, reflecting neurodegeneration and neuroinflammation, while the PCA2 pattern contains elevated A*β*42/40 and low P-tau181/A*β*42, and thus is more AD-specific [20]. In the prior study, individuals who developed dementia or MCI had faster decrease in PCA2 score compared to those who remained cognitively normal during follow-up [20]. We hypothesized that individuals with the highest relative O-3, O-6 and MUFA intake would have lesser progression of AD pathophysiology over time reflected in biomarker levels.

To evaluate whether a reduced burden of cardiovascular disease mediated the association, we included a final model adjusting for a cardio-cerebrovascular risk factor (CVRF) score. This score was previously derived in our cohort based on body mass index and history of heart disease, hypertension and diabetes. We performed a secondary analysis using individual O-3 fatty acids, including eicosapentaenoic (EPA), docosapentaenoic acid (DPA) and docosahexaenoic acid (DHA).

## 2. Methods

### 2.1. Cohort and clinical diagnosis

The Washington Heights-Inwood Columbia Aging Project (WHICAP) is a multiethnic, community-based prospective cohort study about risk factors for dementia. This study was approved by the institutional review boards of Columbia University. All participants provided written informed consent.

The study design has been described in detail elsewhere [21]. Briefly, WHICAP participants are individuals 65 years and older, residing in northern Manhattan, who speak English or Spanish. There have been three major waves of enrollment, beginning in 1992 (93 participants in the current study), 1999 (44 participants), and 2009 (462 participants).

Each visit was comprised of a structured interview of demographics, lifestyle information, medical and neurological history, followed by a standardized physical exam, a neurological exam, and neuropsychiatric testing. Participants completed a 61-item Willett semi-quantitative food frequency questionnaire (SFFQ) (Channing Laboratory, Cambridge, Massachusetts) at their baseline visit only. Race and ethnicity were reported and coded using standardized criteria [22]. At the baseline and each subsequent visit, blood was drawn. Participants returned for follow-up visits every 18 to 24 months.

A diagnostic consensus conference for each visit was attended by at least one neurologist and one neuropsychologist with experience in AD. Using neuropsychological testing and evidence of functional impairment, a clinical diagnosis of probable or possible AD was assigned based on the National Institute on Aging-Alzheimer’s Association criteria [23]. Evaluators were blinded to previous diagnoses for follow-up visits.

### 2.2. Inclusion and exclusion criteria

For the current study, we selected individuals without dementia at baseline who completed the SFFQ at their initial visit and had plasma biomarkers measured during at least two different visits (Supplementary eFigure 1). To be included, participants had to have biomarkers measured at least at 2 or 3 visits. For one participant with 4 visits, the last one was excluded from the analysis (eFigure 1). We excluded 6 participant visits in which there were outliers (|Z-score| > 3) in any of the chosen plasma biomarkers. We excluded 2 participants for outliers at the baseline visit and 1 participant for an insufficient total number of blood samples (<2) after the removal of a follow-up visit which contained an outlier. In total, 10 participant visits were excluded from the analysis (eFigure 1).

### 2.3. Plasma collection and plasma biomarkers measurement

At the baseline and each subsequent visit, blood samples were collected by standard venipuncture in dipotassium ethylenediaminetetraacetic acid tubes, as previously described [12]. Study visits occurred in the morning or afternoon and participants were not required to fast. Briefly, plasma biomarker assays were performed using the single molecule array technology Quanterix Simoa (single molecule array) [22] HD-X platform (Quanterix, Billerica, MA, USA). Samples were diluted and assayed in duplicate using three Quanterix kits: Neurology 3-Plex A (catalog No. 101,995) for A*β*42 and A*β*40; P-tau181 V2 Advantage (catalog No. 103,714) for Tau phosphorylated at threonine 181 (P-tau181); and Neurology 2-Plex B (catalog No. 103,520) for NfL and GFAP. Quantification functional lower limits for these analytes were 2.7pg/mL for A*β*40, 0.6 pg/mL for A*β*42, 0.3 pg/mL for P-tau181, 0.8 pg/mL for NfL, and 16.6 pg/mL for GFAP. Mean sample coefficient of variation for each biomarker, calculated from the duplicate samples, was less than 5%. Ratios of A*β*42/A*β*40 and P-tau181/A*β*42 were calculated.

### 2.4. Nutrients

The SFFQs have been used and validated in the elderly [24] and in our cohort [25]. Daily intake of nutrients in miligrams was computed by multiplying the consumption frequency of each food by the content of the specified nutrient [26]. We excluded nutrient intake from supplements to focus on the effects of naturally occurring dietary sources [27], minimize variability from inconsistent supplement use, and avoid potential biases such as reverse causation and misreporting. For this analysis, we used the residuals of the linear regression of total energy intake on the intake of each nutrient to avoid confounding by total energy intake. The residual of each nutrient was further categorized into tertiles.

A score indicating adherence to the Mediterranean diet (MeDi) [28] was used as an indicator of overall dietary quality. One point each was given for higher-than-median intake of food in six beneficial categories (fruits, vegetables, legumes, cereals, and fish). One point was given for higher-than-median ratio of monounsaturated to saturated fats. One point each was given for lower-than-median intake of two detrimental food categories (meat, dairy). One point was given for mild to moderate alcohol consumption (0–30*g*/day)). Overall, the MeDi score ranged 0–9, with a higher score indicating better adherence.

### 2.5. Covariates

The CVRF score was created in the WHICAP cohort as previously described [29], using principal component analysis of body mass index (measured at in person visits) and self-reported hypertension, diabetes and heart disease. The validity of self-reported vascular risk factors has been confirmed in our cohort [30]. Education was recorded in years based on self-report. We used *APOE ε*4 genotype dichotomously as the presence (either 1 or 2) or absence of *ε*4 alleles. Charlson Comorbidity Index [31] was assessed at the initial visit only.

### 2.6. Statistical analyses

We compared descriptive variables across nutrient intake tertiles (low, middle, high) using one-way ANOVA for continuous variables and Chi-Square for categorical variables. For variables that differed according to nutrient intake tertile (*p* < 0.05), we performed a post-hoc analysis to determine which levels of intake (low, middle or high) differed from one another. We used Tukey Honestly Significant Differences for continuous variables and Chi Square for categorical variables.

In cross-sectional analysis, we evaluated the association between nutrient intake tertile and biomarker concentrations at baseline using Generalized Linear Models. In longitudinal analysis, we evaluated the effect of the interaction between nutrient intake tertiles and the passage of time (in number of visits) on biomarker concentrations, using Generalized Estimating Equations (GEE) with an autoregressive correlation structure. Participants with missing values were eliminated from the models. For both cross-sectional and longitudinal analyses, we included three models each. Model 1 was adjusted for total energy intake only. Model 2 was adjusted for total energy intake, age at baseline, sex, ethnicity, education and storage time. For Model 2, we included a trend analysis in which nutrient intake tertile was coded as a continuous variable [1–3]. Model 3 was adjusted for Model 2 covariates and CVRF score.

In secondary analysis, we evaluated the effect of the individual O-3 PUFAs EPA, DHA, and DPA on longitudinal biomarker concentrations. We evaluated the interaction sex ^∗^ nutrient intake tertile on baseline biomarker concentration. We also evaluated whether the associations we identified in the primary analysis (Model 2) remained significant after adjusting for MeDi adherence and the presence of *APOE ε4*, and after adjusting for the Charlson Comorbidity Index. We repeated Model 1 and Model 2 of the primary analysis using time in years. Lastly, we repeated the analysis with mixed effects models with random effects for repeated measures.

All p-values for trend and continuous analyses were adjusted for multiple comparisons using the Benjamini & Hochberg false discovery rate (FDR) method. Results were considered to be statistically significant when FDR-adjusted P value was < 0.05.

## 3. Results

### 3.1. Study participants

599 participants were included in the current study (eFigure 1). 52 participants had biomarkers measured at 2 visits (one baseline and one follow up visit), and 547 participants had biomarkers measured at 3 visits. Follow-up time ranged from 1.5 to 22.9 years, with a mean of 7.0 years. Participants had attained on average 11.3 years of education at baseline and had a MeDi adherence score of 4.5 (Table 1). Twenty-five percent had at least one APOE ε4 allele, and 13% developed dementia during the follow up period (Table 1). The mean Charlson Comorbidity Index score was 2.2 (Table 1).

### 3.2. Descriptive analyses

At baseline, participants with O-3, O-6 and MUFA intakes in the high tertiles were younger (Table 1, Supplementary eTables 1–2). Those with intake in higher tertiles of all three fatty acids had more years of education (Table 1, Supplementary eTables 1–2). O-6 PUFA and MUFA intake tertile varied by ethnicity (Supplementary eTables 1–2), with non-Hispanic White and non-Hispanic Black individuals with intake in higher tertiles than Hispanic individuals. Individuals with O-3 intake in the high tertile consumed less total energy than those with intake in the low tertile (Table 1), whereas there was not a consistent trend for O-6 or MUFA intake tertile (eTables 1–2). Participants with high tertile O-3 intakes had higher MeDi adherence (Table 1), but those with high tertile MUFA intakes had lower adherence compared to those with low tertile intakes (eTable 2). Participants did not vary in their MeDi adherence by O-6 tertile (eTable 2). About 0.5%, 40%, and 100% of the participants with O-3 intakes in the low, middle, and high tertiles consumed 2 or more portions of fish per week (Table 1). Those with high tertile O-6 and MUFA intakes were less likely to develop dementia at a follow up visit compared to those with low tertile intakes (Supplementary eTables 1–2). Those with O-6 intakes in the high tertile had lower BMI and lower Charlson Comorbidity Index compared to those with intakes in the low tertile (eTable 2).

### 3.3. Cross-sectional analyses

**Fig. 1.**
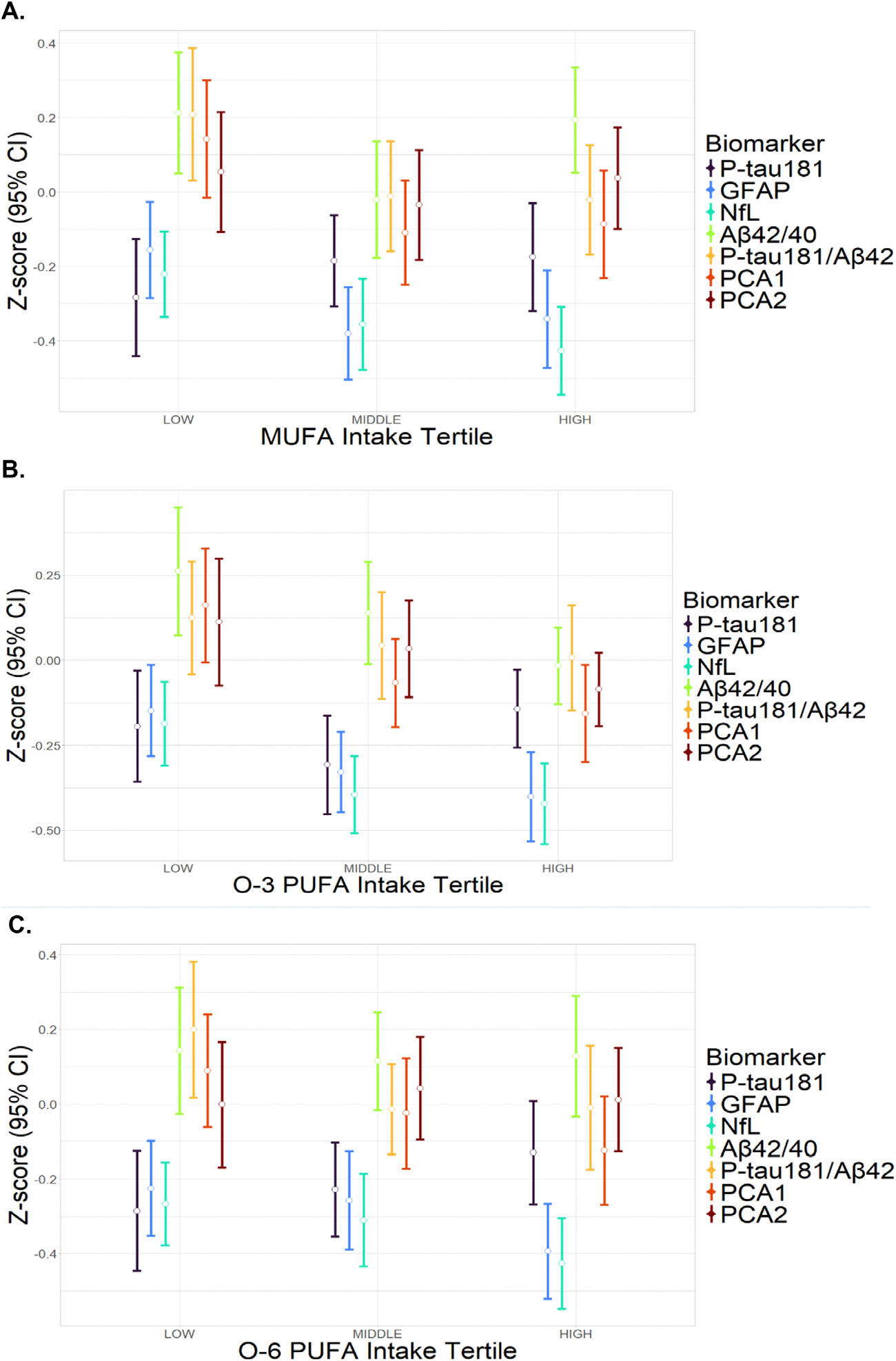
Baseline biomarker levels by nutrient intake tertile. Abbreviations (units): O-3, omega-3: O-6, omega-6: PUFA, polyunsaturated fatty acid: MUFA, monounsaturated fatty acid: P-tau181, phosphorylated tau 181 level (pg/ml, log10-transformed): A*β*42/40, amyloid-beta 42/40 ratio (log10-transformed): GFAP, glial fibrillary acidic protein level (pg/ml, log10-transformed): NfL, neurofilament light chain level (pg/ml, log10-transformed): PCA1, first principal component (derived from the latter four biomarkers): PCA2, second principal component. Generalized linear models were used to evaluate the association of nutrient intake (high and middle compared to low tertiles) with baseline biomarker level. Missing values excluded. *N* = 593 for A*β*42/40, *N* = 598 for GFAP and NfL, *N* = 597 for P-tau181, *N* = 593 for P-tau181/ A*β*42, *N* = 520 for PCAs. The model was adjusted for total calorie intake, age, sex, ethnicity, education, and storage time. No comparisons were statistically significant after correction for 21 comparisons using the False Discovery Rate method.

At baseline, there were numeric differences, although without statistical significance in trend analysis, in biomarker levels by nutrient intake tertiles, with higher O-3 having lower levels of GFAP, NfL, and PCA1 (Fig. 1, eTable 3).

### 3.4. Longitudinal analyses

In our study participants, P-tau181 (*β*= 0.048 log-transformed pg/ml per visit, p-adjusted < 0.001), GFAP (*β*= 0.063, p-adjusted < 0.001) and NfL levels (*b* = 0.076, p-adjusted < 0.001) increased across subsequent visits while A*β*42/40 ratio (*β*= −0.153, p-adjusted < 0.001) and P-tau181/A*β*42 ratio (*β*= −0.017, p-adjusted = 0.024) decreased. Neither PCA1 (*β*= 0.248, p-adjusted = 0.20) nor PCA2 score (*β*= −0.007, p-adjusted = 0.746) changed significantly over time.

**Fig. 2.**
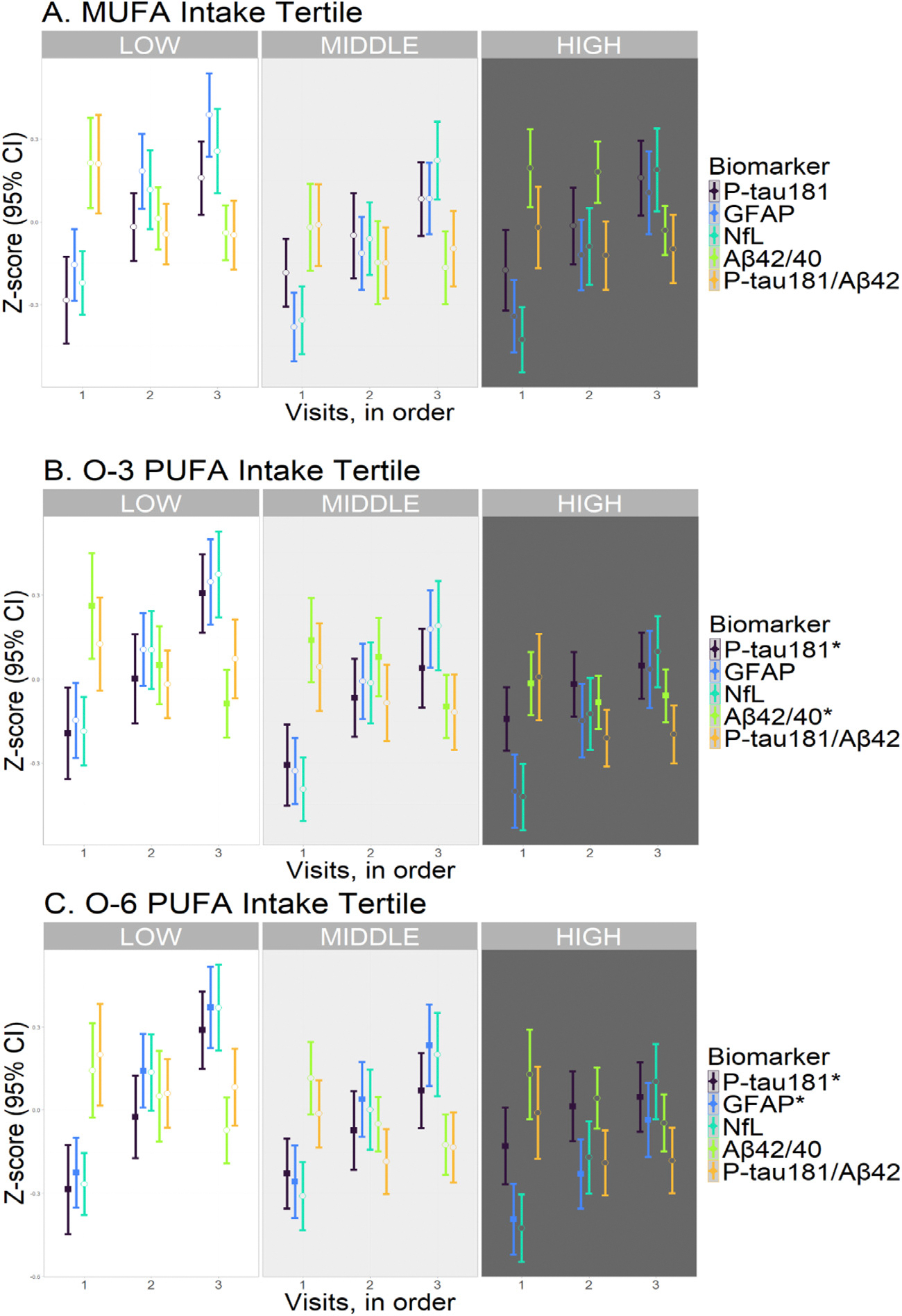
Longitudinal biomarker levels by nutrient intake tertile. Abbreviations (units): O-3, omega-3: O-6, omega-6: PUFA, polyunsaturated fatty acid: MUFA, monounsaturated fatty acid: P-tau181, phosphorylated tau 181 level (pg/ml, log10-transformed): A*β*42/40, amyloid-beta 42/40 ratio (log10-transformed): GFAP, glial fibrillary acidic protein level (pg/ml, log10-transformed): NfL, neurofilament light chain level (pg/ml, log10-transformed). Generalized estimating equations (GEE) were used to evaluate the association of nutrient intake (high and middle compared to low tertiles) with change in biomarker level over time (in visits). The results of model 2, adjusted for total energy intake, age, sex, ethnicity, education and storage time, are shown (*N* = 598). ^∗^ False discovery rate (FDR) adjusted P-values from trend analysis <0.05, corresponding to filled squares in the graph. Open circles denote non-significant differences.

**Fig. 3.**
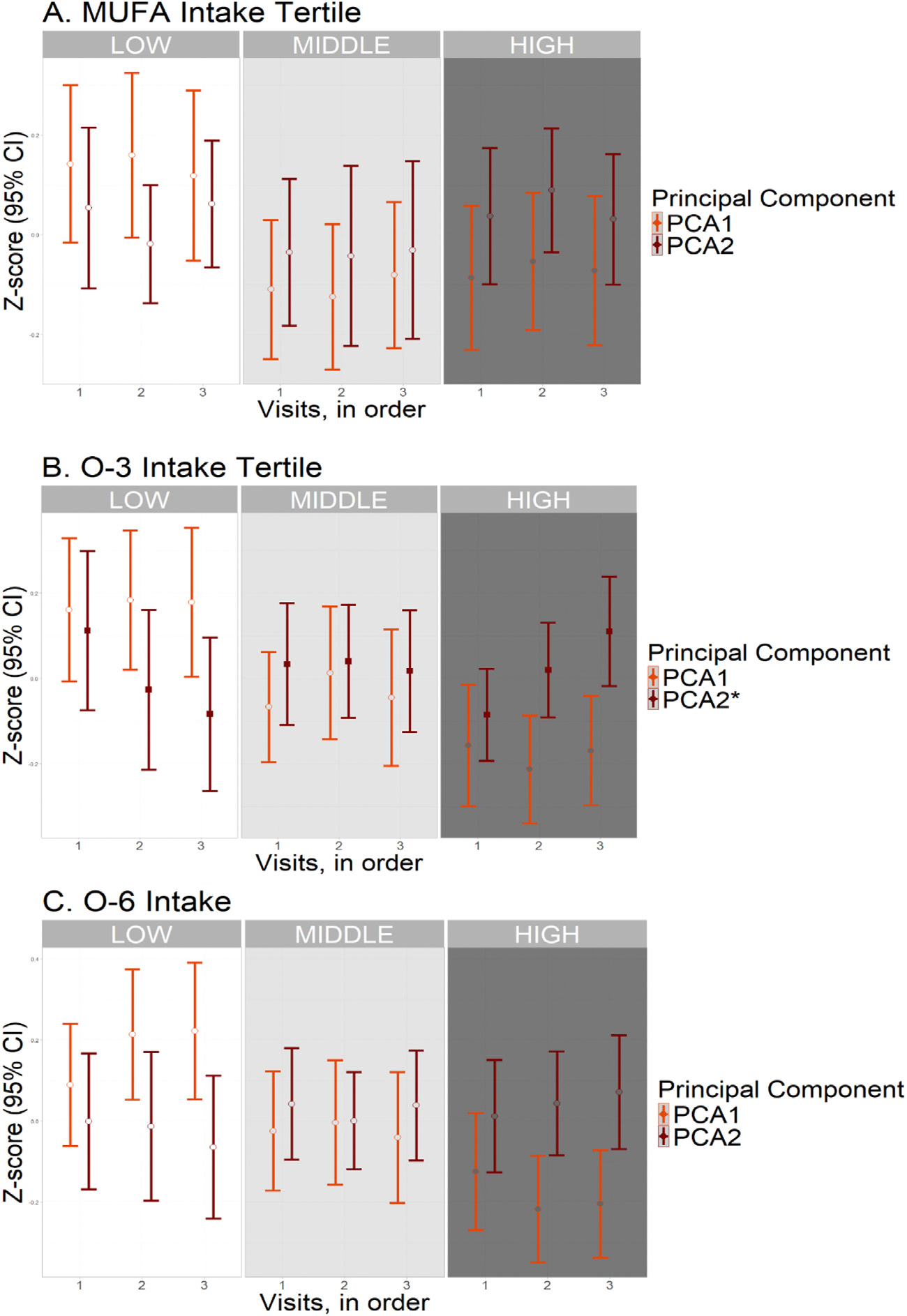
Longitudinal principal component scores by nutrient intake tertiles. Abbreviations (units): O-3, omega-3: O-6, omega-6: PUFA, polyunsaturated fatty acid: MUFA, monounsaturated fatty acid: PCA1, first principal component (derived from the latter four biomarkers): PCA2, second principal component. Generalized estimating equations (GEE) were used to evaluate the association of nutrient intake (high and middle compared to low tertiles) with change in principal component score over time (in visits). The results of model 2, adjusted for total energy intake, age, sex, ethnicity, education and storage time, are shown (*N* = 598). ^∗^ False discovery rate (FDR) adjusted P-values from trend analysis <0.05, corresponding to filled squares in the graph. Open circles denote non-significant differences.

Individuals with high tertile O-3 intakes had a lesser increase in P-tau181 level (*β*= −0.037 log-transformed pg/ml, *p* = 0.001, p-trend = 0.006), and lesser decrease in A*β*42/40 ratio (*β*= 0.022, *p* = 0.003, p-trend = 0.013) across subsequent visits compared to those with low tertile intakes (Fig. 2, Table 2). They also had a lesser decrease in PCA2 score (*b* = 0.221, *p* < 0.001, p-trend = 0.001) over time (Fig. 3, Table 2). After adjusting for CVRF score, the associations of O-3 PUFA tertile with PCA2 score (*β*= 0.168, *p* = 0.010) and P-tau181 level (*β*= −0.030, *p* = 0.027) persisted. However, the association with A*β*42/40 ratio was no longer significant (*β*= 0.013, *p* = 0.097) (Table 2).

Individuals with O-6 intakes in middle (*β*= −0.037, *p* = 0.003) and high (*β*= −0.050, *p* < 0.001) tertiles had a lesser increase in P-tau181 concentration over time, compared to those with intakes in the low tertile (p-trend < 0.001) (Fig. 2, Table 2). These associations remained significant after adjusting for CVRF score (middle, *β*= −0.033, *p* = 0.025; high, *β*= −0.046, *p* = 0.002) (Table 2). Those with O-6 intakes in the high tertile also had a lesser increase in GFAP level (*β*= −0.028, *p* = 0.002, p-trend = 0.008) (Fig. 2, Table 2), even after adjusting for CVRF score (*β*= −0.028, *p* = 0.009) (Table 2).

### 3.5. Secondary analysis comparing subtypes of O-3 PUFAs

Compared to participants with low tertile intakes of EPA, those in with high tertile intakes had a lesser increase in P-tau181 level (*β*= −0.033, *p* = 0.004, p-trend = 0.016) and a lesser decrease in PCA2 score over time (*β*= 0.205, *p* < 0.001, *p* = 0.002) (eTable 4). Those with middle (*β*= −0.028, *p* = 0.034) and high (*β*= −0.037, *p* = 0.004) tertile DPA intakes had a lesser rise in P-tau181 compared to those with low tertile intakes (p-trend = 0.016) (eTable 4). Those with high tertile intakes had a lesser decline in A*β*42/40 (*β*= 0.023, *p* = 0.001, p-trend = 0.009) compared to those with low tertile intakes (eTable 4). Those with middle (*β*= 0.155, *p* = 0.010) and high (*β*= 0.226, *p* < 0.001) tertile DPA intakes had a lesser decrease in PCA2 score over time (p-trend = 0.001), compared to those with low tertile intakes (eTable 4). Lastly, those with high tertile DHA intakes had a lesser rise in P-tau181 level (*β*= −0.030, *p* = 0.009, p-trend = 0.027) and a lesser decrease in PCA2 score (*β*= 0.169, *p* = 0.003, p-trend = 0.015), compared to those with low tertile intakes (eTable 4).

### 3.6. Sensitivity analyses

Models 1 and 2 of the primary analysis were repeated with time measured in years and (residuals of) continuous nutrient intake in mg/day. Higher O-3 intake was associated with a lesser change in P-tau 181 level and PCA2 score over time (eTable 5). However, O-3 PUFA intake was not associated with change in A*β*42/40 ratio, and O-6 PUFA intake was not significantly associated with biomarkers (eTable 5). Repeating the analysis using mixed effects models with random effects for repeated measures yielded consistent results (eTable 6), and all associations identified in Model 2 of the original analysis (Table 2) remained significant.

Higher MeDi adherence was associated with lower baseline PCA1 score (*p* = 0.046) when adjusting for total energy intake only, but not after adjusting for other covariates, including baseline age, sex, ethnicity, education and storage time (eTable 7). Higher MeDi score was numerically associated with lesser decrease in PCA2 score over time, but this was not statistically significant after adjustment for 7 comparisons (eTable 7). The differences in longitudinal change in biomarkers in the primary analysis (Model 2, Table 2) persisted after additionally adjusting for MeDi score and *APOE* ε4 status (data not shown).

When excluding participants with incident dementia, we found similar results in longitudinal analysis, except that the middle (*β*= −0.101, *p* = 0.035) and high (*β*= −0.130, *p* = 0.004) tertile O-6 intakes were additionally associated with lesser increase in PCA1 score over time compared to low tertile intake (p-trend = 0.022) (Supplementary eTable 8). Adjustment for the Charlson Comorbidity Index did not alter the results (eTable 9). Evaluating the interaction sex ^∗^ nutrient tertile on baseline biomarker concentrations did not show a significant difference between men and women (eTable 10).

## 4. Discussion

In a multi-ethnic community-based cohort of older adults without baseline dementia, individuals with high tertile O-3 intake had a lesser progression of AD-related biomarkers across subsequent visits compared to those with low intake, including a lesser decrease in PCA2 score, a lesser increase in P-tau181 level and a lesser decrease in A*β*42/40 ratio. In addition, those with high O-6 intake tertile had a lesser increase in P-tau181 and GFAP levels compared to those with low intake tertile. Most of these relationships persisted after adjusting for cardiovascular risk factor score.

The results suggest that individuals with higher relative intakes of both O-3 and O-6 PUFAs had slower progression of AD pathophysiology over time. This protective association corresponded mostly to AD-specific changes (A*β*42/40 and P-tau181) rather than less specific markers of nervous system injury (GFAP and NfL). This distinction underscores the possibility that PUFA, especially O-3, might modulate pathological processes central to AD, such as amyloid plaque formation and tau pathology, rather than general neuronal damage or inflammation. Associations of each of the individual O-3 PUFAs (EPA, DPA and DHA) with biomarkers were similar to those identified for O-3 in the primary analysis, and they suggest that the individual PUFAs have similar relationships with AD biomarkers. It is unclear why individuals with the highest O-3 intake had a longitudinal increase in PCA2 score, increase in A*β*42/40 ratio (component 1 of PCA2 score) and decrease P-tau181, but no significant change in P-tau181/A*β*42 (component 2 of PCA2 score). However, the numeric change in P-tau181/A*β*42 associated with high tertile O-3 intake was in the expected direction. Further studies could explore the temporal sequence of biomarker changes in response to O-3 intake.

We hypothesized that dietary intake of key nutrients would reduce risk of AD, reflected in a lesser increase in biomarker levels, via a reduced burden of vascular risk factors. A growing body of literature suggests that vascular comorbidities may influence the levels of plasma biomarkers [32–34]. In one biracial cohort, individuals with diabetes had lower GFAP levels, those with history of stroke had higher NfL, and those with chronic kidney disease had higher NfL and lower P-tau181 [32]. Other studies have shown variation in white matter hyperintensity, a marker for microvascular disease, by P-tau181, P-tau217 [35] and GFAP levels [36]. EPA and DHA were associated with reduced risk of cardiovascular disease in prospective cohort studies [37]. In prospective cohorts and randomized controlled trials, plant-based oils enriched in the O-6 PUFA linoleic acid largely seem protective against coronary artery disease [37]. Nonetheless, several of the associations we identified between O-3 and O-6 intake and change in biomarkers persisted after adjusting for CVRF score, suggesting the nutrients might be associated with the AD-related pathological markers through pathways independent of vascular risk.

There are several studies linking intake of O-3 PUFAs with a lesser burden of amyloid pathology. One study showed that higher intake of O-3, vitamin B12 and vitamin D was associated with lower positron emission tomography (PET) amyloid burden in cognitively normal participants [17]. Another study reported higher O-3 intake was associated with lower level of cerebrospinal fluid (CSF) AD biomarkers in participants with mild AD, although inconsistent results on O-3 and CSF biomarkers also exist [18,19]. These suggest that higher O-3 intake may protect against amyloid pathological changes preceding and in early AD. We found that PUFAs were not associated with P-tau181 at baseline in the cross-sectional analysis but predicted less increase in phospho-tau longitudinally. One possible explanation of this is that, at baseline, the tau pathology might still be minimal in the study population, making it difficult to detect significant associations. In contrast, the change over many years of follow-up may better capture individual differences in tau dynamics. An alternative explanation is that cross-sectional P-tau levels are likely influenced by various individual factors such as age, metabolism, genetic predisposition, and comorbidities. In comparison, longitudinal changes in P-tau reflect within-individual trajectories and may be less affected by these external sources of variability.

There are few other observational studies on the influence of fatty acids on blood-based AD biomarkers, with varying results. In a cross-sectional study in our cohort, individuals with higher O-3 consumption had reduced plasma A*β*42 [14]. A multidomain interventional study including an O-3 supplement improved composite cognitive scores in non-demented individuals with low plasma A*β*42/40 ratio indicating risk of AD [16]. In a randomized controlled trial, treatment with a nutritional supplement containing EPA and DHA O-3 fatty acids did not affect P-tau181 or GFAP levels after 1 year compared to placebo [15].

Biological mechanisms that might be responsible for the association between O-3 and AD biomarkers are unclear. PUFAs are critical components of neuronal membranes, contributing to membrane fluidity and proper functioning of membrane-bound ion channels [38]. However, membrane PUFAs are also highly susceptible to lipid peroxidation due to their double bonds. Peroxidized PUFAs can initiate ferroptotic cell death by iron dependent enzymes [39], which leads to release of pro-inflammatory signals and activates microglia and astrocytes, contributing to AD. In a study of hypertensive older adults, oxidized products of O-3 and O-6 were associated with increased white matter hyperintensity and poorer executive function [40]. However, adequate levels of O-3 PUFAs have been shown to reduce oxidative stress by several mechanisms. They support synthesis of glutathione, which detoxifies lipid peroxides, and they improve expression of antioxidant enzymes, like glutathione peroxidase 4 [39]. Lastly, they promote the production of specialized pro-resolving mediators, such as resolvins and protectins, which help counteract peroxidation damage [41]. Overall, O-3 PUFAs may exert neuroprotective effects at least partly by reducing inflammation and oxidative stress.

Our study has several limitations. Dietary intake, which we assessed once at the initial visit, may have changed over time. However, we previously showed that dietary habits appear stable over time within individuals [25,28]. Misclassification of dietary data may be present, as the standard SFFQ used in our study might not fully capture all foods consumed by our multi-ethnic, elderly population, particularly the culturally specific or traditional foods. Given the observational nature of our study, we cannot make definite conclusions about whether intake of these key nutrients caused a lesser progression in biomarkers. It is possible that there was residual confounding of demographic and social factors with nutrient intake and the progression of AD pathophysiology. In addition, many individuals were missing PCA scores and/or CVRF score.

Plasma biomarkers, minimally invasive way to monitor AD-related brain changes, may provide valuable insight into the influence of diet on progression of AD pathophysiology before the onset of cognitive symptoms. This study suggests that individuals with calculated high tertiles of O-3 and O-6 PUFAs from food have lesser progression of AD biomarkers over time compared to those with low intakes. It also suggests that this protective association may occur separately from the effects of nutrition on cardiovascular risk. More studies are needed to understand this association, and to identify whether there might be individuals that could benefit from increased O-3 and O-6 intake, in the broad strategy to impact AD.

## Supplementary Material

supp

## Figures and Tables

**Table 1:** Summary of participants by O-3 PUFA intake tertile.

Characteristic*	O-3 PUFA Intake Tertile				p-value*
	LOW *N* = 200^†^	MIDDLE *N* = 199^†^	HIGH *N* = 200^†^	Overall *N* = 599^‡^	

O-3 PUFA, mg/d	86.4 (77.6)	200 (162)	463 (454)	250 (323)	< 0.001
Age	74.5 (5.88)	74.6 (6.08)	72.3 (4.91)	73.8 (5.74)	< 0.001
Women	143 (72%)	135 (68%)	139 (70%)	417 (70%)	0.728
Men	57 (29%)	64 (32%)	61 (31%)	182 (30%)	
Ethnicity					0.130
Non-Hispanic White	53 (27%)	52 (26%)	62 (31%)	167 (28%)	
Non-Hispanic Black	42 (21%)	50 (25%)	58 (29%)	150 (25%)	
Hispanic	105 (53%)	97 (49%)	80 (40%)	282 (47%)	
Energy intake, kcal	1.47 (0.46)	1.28 (0.453)	1.26 (0.397)	1.34 (0.447)	< 0.001
Consumption of 2+ portions of fish, weekly	1 (0.5%)	80 (40%)	200 (100%)	281 (48%)	< 0.001
O-6 PUFA, mg/d	7880 (4020)	6640 (3530)	7420 (3860)	7310 (3840)	0.004
MUFA, mg/d	15,600 (7050)	13,100 (6420)	13,800 (6440)	14,200 (6710)	< 0.001
MeDi score	3.8 (1.56)	4.54 (1.73)	5.14 (1.35)	4.5 (1.64)	< 0.001
Education, years	10.3 (5.42)	10.9 (5.1)	12.6 (5.19)	11.3 (5.33)	< 0.001
APOE *ε*4 allele^§^	50 (25%)	54 (27%)	47 (24%)	151 (25%)	0.718
BMI (kg/m^2)	27.9 (4.36)	27 (4%)	27.8 (4.63)	27.5 (4.36)	0.123
Diabetes mellitus	64 (32%)	62 (31%)	73 (37%)	199 (33%)	0.476
Heart disease	96 (48%)	89 (45%)	75 (38%)	260 (43%)	0.095
Hypertension	173 (87%)	162 (81%)	168 (84%)	503 (84%)	0.382
Charlson Comorbidity Index	2.21 (1.47)	2.19 (1.48)	2.18 (1.53)	2.2 (1.49)	0.984
Incident dementia	32 (16%)	27 (14%)	20 (10%)	79 (13%)	0.204
P-tau181 level (pg/ml)	2.78 (2.53)	2.44 (1.36)	2.66 (2.15)	2.63 (2.07)	0.179
GFAP level (pg/ml)	185 (97.8)	163 (82.5)	162 (121)	170 (102)	0.034
NfL level (pg/ml)	23.9 (13.2)	20.9 (11.4)	20.8 (11.4)	21.8 (12.1)	0.020
P-tau181/A*β*42 ratio	0.443 (0.999)	0.407 (1.1)	0.472 (1.8)	0.441 (1.34)	0.893
A*β*42/40 ratio	0.0555 (0.0656)	0.0484 (0.0239)	0.0447 (0.0179)	0.0495 (0.0419)	0.032
PCA1	0.161 (1.11)	−0.0668 (0.868)	−0.157 (0.974)	−0.0249 (0.992)	0.017
PCA2	0.112 (1.23)	0.0336 (0.96)	−0.0855 (0.737)	0.0176 (0.992)	0.146

Abbreviations (units): O-3, omega-3 (mg/day): O-6, omega-6 (mg/day): PUFA, polyunsaturated fatty acid (mg/day): MUFA, monounsaturated fatty acid (mg/day): APOE *ε*4, apolipoprotein E epsilon 4 allele: MeDi, Mediterranean diet score: BMI, body mass index (kg/m^2): P-tau181, phosphorylated tau 181 (pg/ml): A*β*42/40, amyloid-beta 42/40 ratio: GFAP, glial fibrillary acidic protein (pg/ml): NfL, neurofilament light chain (pg/ml): PCA1, first principal component (derived from the latter four biomarkers): PCA2, second principal component.

* Missing values were excluded from analysis. Fish intake available for 591 individuals, education available for 598 individuals, APOE *ε*4 available for 598 individuals, MeDi score available for 591 individuals, BMI available for 522 individuals, Charlson Comorbidity Index available for 597 individuals, P-tau181 available for 598 individuals, A*β*42/40 available for 594 individuals.

† Mean (SD); n (%).

‡ One-way analysis of means (not assuming equal variances); Pearson’s Chi-squared test.

**Table 2:** Association of baseline nutrient intake tertile with longitudinal biomarker changes.

Intake	Biomarker	Model 1*		Model 2^†^		Trend^‡^	Model 3^§^	
		Estimate	P	Estimate	P	Corrected P	Estimate	P

**MUFA intake tertile**								
HIGH	P-tau181	−0.019	0.159	−0.019	0.149	0.315	−0.026	0.113
MIDDLE		**−0.026**	**0.027**	**−0.026**	**0.024**		−0.020	0.157
HIGH	A*β*42/40	0.006	0.397	0.006	0.387	0.575	0.013	0.113
MIDDLE		0.009	0.240	0.008	0.281		**0.017**	**0.039**
HIGH	P-tau181/A*β*42	0.025	0.146	0.025	0.143	0.315	0.035	0.089
MIDDLE		0.025	0.160	0.024	0.172		0.040	0.051
HIGH	GFAP	−0.014	0.144	−0.014	0.136	0.315	−0.016	0.166
MIDDLE		−0.013	0.161	−0.013	0.150		−0.010	0.376
HIGH	NfL	0.015	0.160	0.015	0.172	0.315	0.012	0.383
MIDDLE		0.006	0.572	0.006	0.594		0.013	0.287
HIGH	PCA1	0.022	0.612	0.023	0.601	0.666	0.034	0.534
MIDDLE		0.016	0.724	0.013	0.774		0.066	0.219
HIGH	PCA2	0.016	0.773	0.016	0.767	0.771	0.040	0.541
MIDDLE		0.022	0.701	0.016	0.779		0.045	0.483
**O-3 PUFA intake tertile**								
HIGH	P-tau181	**−0.037**	**0.001**	**−0.037**	**0.001**	**0.006**	**−0.030**	**0.027**
MIDDLE		−0.014	0.308	−0.014	0.315		−0.001	0.960
HIGH	A*β*42/40	**0.023**	**0.002**	**0.022**	**0.003**	**0.013**	0.013	0.097
MIDDLE		0.010	0.212	0.010	0.209		0.004	0.631
HIGH	P-tau181/A*β*42	−0.025	0.129	−0.026	0.119	0.315	−0.028	0.163
MIDDLE		−0.015	0.418	−0.014	0.444		−0.010	0.638
HIGH	GFAP	−0.006	0.538	−0.007	0.502	0.586	−0.003	0.830
MIDDLE		0.004	0.700	0.004	0.683		0.006	0.621
HIGH	NfL	−0.003	0.775	−0.003	0.738	0.771	0.010	0.425
MIDDLE		0.008	0.470	0.009	0.449		0.018	0.180
HIGH	PCA1	−0.026	0.573	−0.031	0.492	0.586	0.004	0.942
MIDDLE		0.005	0.908	0.007	0.882		0.020	0.726
HIGH	PCA2	**0.225**	**0.000**	**0.221**	**0.000**	**0.001**	**0.168**	**0.010**
MIDDLE		0.112	0.064	0.113	0.060		0.065	0.348
**O-6 PUFA intake tertile**								
HIGH	P-tau181	**−0.049**	**0.000**	**−0.050**	**0.000**	**0.001**	**−0.047**	**0.002**
MIDDLE		**−0.036**	**0.004**	**−0.037**	**0.003**		**−0.033**	**0.025**
HIGH	A*β*42/40	0.007	0.395	0.006	0.455	0.586	0.011	0.187
MIDDLE		0.001	0.872	0.001	0.899		0.002	0.820
HIGH	P-tau181/A*β*42	−0.012	0.501	−0.013	0.475	0.586	−0.005	0.801
MIDDLE		−0.006	0.712	−0.006	0.709		−0.006	0.749
HIGH	GFAP	**−0.027**	**0.003**	**−0.028**	**0.002**	**0.009**	**−0.028**	**0.009**
MIDDLE		−0.015	0.123	−0.015	0.108		−0.011	0.331
HIGH	NfL	−0.012	0.274	−0.012	0.242	0.396	−0.006	0.633
MIDDLE		−0.015	0.150	−0.016	0.131		−0.007	0.557
HIGH	PCA1	**−0.097**	**0.025**	**−0.102**	**0.019**	0.070	−0.071	0.170
MIDDLE		−0.070	0.135	−0.071	0.130		−0.017	0.761
HIGH	PCA2	0.082	0.153	0.077	0.180	0.315	0.090	0.169
MIDDLE		0.048	0.393	0.046	0.412		0.037	0.588

Abbreviations (units): O-3, omega-3: O-6, omega-6: PUFA, polyunsaturated fatty acid: MUFA, monounsaturated fatty acid: P-tau181, phosphorylated tau 181 level (pg/ml, log10-transformed): A*β*42/40, amyloid-beta 42/40 ratio (log10-transformed): GFAP, glial fibrillary acidic protein level (pg/ml, log10-transformed): NfL, neurofilament light chain level (pg/ml, log10-transformed): PCA1, first principal component (derived from the latter four biomarkers): PCA2, second principal component.

Generalized estimating equations (GEE) were used to evaluate the association of nutrient intake (high and middle compared to low tertiles) with change in biomarker level over time (in visits). Missing values excluded. P-values < 0.05 shown in bold.

* Model 1 was adjusted for total energy intake only (*N* = 599 for biomarkers, *N* = 527 for PCAs).

† Model 2 was adjusted for total energy intake, age, sex, ethnicity, education, and storage time (*N* = 598 for biomarkers, *N* = 526 for PCAs).

‡ A trend analysis of Model 2 was performed, using nutrient intake tertile as a continuous integer [1–3]. P-values corrected for 21 comparisons using the False Discovery Rate (FDR) are shown.

§ Model 3 was adjusted for Model 2 covariates plus cardiovascular risk factor (CVRF) score (*N* = 431 for biomarkers, *N* = 374 for PCAs).
