# Supplementary material for: Association of dietary fatty acids with longitudinal change in plasma-based biomarkers of Alzheimer's disease
Source: J Prev Alzheimers Dis. 2025 Mar 18;12(5):100117. doi: 10.1016/j.tjpad.2025.100117 (PMC12094269; doi:10.1016/j.tjpad.2025.100117)
Supplement: Supplementary file 1 [file mmc1.docx]

**Supplementary Files**

**Contents**

eFigure 1. Flowchart with subject selection.

eTable 1. Summary of participants by baseline MUFA intake tertile.

eTable 2. Summary of participants by baseline O-6 PUFA intake tertile.

eTable 3. Baseline biomarker levels by nutrient intake tertiles.

eTable 4. Longitudinal change of biomarker levels by intake tertiles of subtypes of O-3 PUFAs.

eTable 5. Longitudinal change of biomarker over time (in years) by nutrient intake (in mg/day)

eTable 6. Longitudinal change of biomarker levels by nutrient intake tertile using mixed effects models.

eTable 7. Baseline and longitudinal association between Mediterranean diet score and biomarkers

eTable 8. Association of baseline nutrient intake with longitudinal biomarker levels, after excluding incident dementia.

eTable 9. Longitudinal change in biomarkers by baseline nutrient intake tertile, additionally adjusted for the Charlson Comorbidity Index.

eTable 10. Interaction of nutrient intake tertile and sex on biomarker levels at baseline.

**eFigure 1.** Flowchart with exclusion criteria.


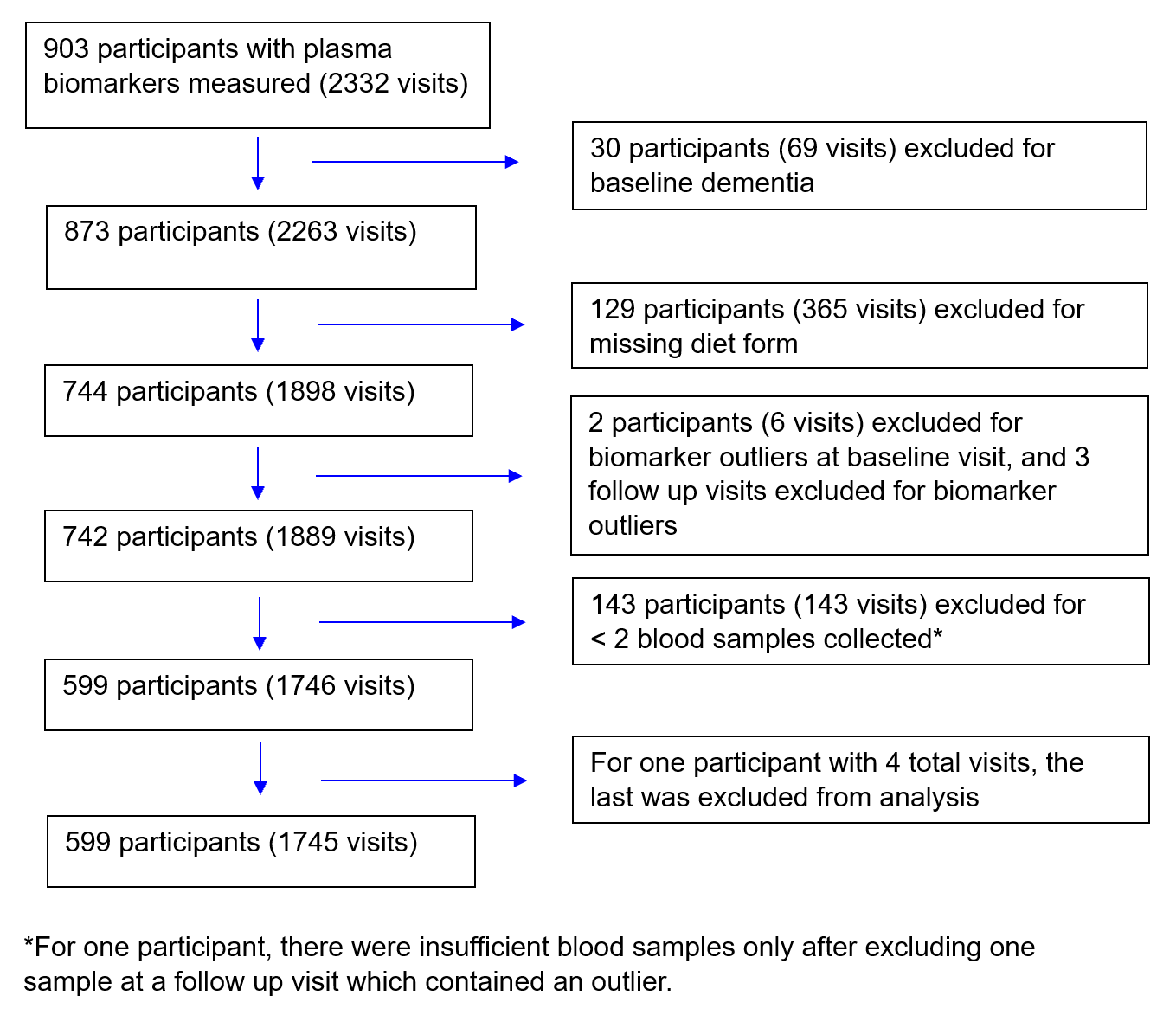


*This includes one participant who was excluded due to insufficient blood samples (<2) after removing a follow-up visit which contained an outlier. In total,10 participant visits were excluded from the analysis due to outliers.

**eTable 1.** Summary of participants by baseline MUFA intake tertile.

|  | **MUFA Intake Tertile** | | | |  |
| --- | --- | --- | --- | --- | --- |
| **Characteristic**^*^ | **LOW**  N = 200^†^ | **MIDDLE**  N = 199^†^ | **HIGH**  N = 200^†^ | **Overall**  N = 599^†^ | **p-value**^‡^ |
| MUFA, mg/d | 10800 (4580) | 12800 (5200) | 18900 (7170) | 14200 (6710) | <0.001 |
| Age | 75.4 (6.41) | 73.4 (5.52) | 72.5 (4.77) | 73.8 (5.74) | <0.001 |
| Women | 144 (72%) | 139 (70%) | 134 (67%) | 417 (70%) | 0.552 |
| Men | 56 (28%) | 60 (30%) | 66 (33%) | 182 (30%) |  |
| Ethnicity |  |  |  |  | <0.001 |
| Non-Hispanic White | 35 (18%) | 58 (29%) | 74 (37%) | 167 (28%) |  |
| Non-Hispanic Black | 28 (14%) | 46 (23%) | 76 (38%) | 150 (25%) |  |
| Hispanic | 137 (69%) | 95 (48%) | 50 (25%) | 282 (47%) |  |
| Energy intake, kcal | 1.39 (0.414) | 1.23 (0.413) | 1.39 (0.493) | 1.34 (0.447) | <0.001 |
| Consumption of 2+ portions of fish, weekly | 86 (44%) | 99 (51%) | 96 (48%) | 281 (48%) | 0.376 |
| O-3 PUFA, mg/d | 209 (222) | 249 (247) | 291 (447) | 250 (323) | 0.041 |
| O-6 PUFA, mg/d | 5700 (2510) | 6640 (2830) | 9600 (4660) | 7310 (3840) | <0.001 |
| MeDi score | 5.23 (1.57) | 4.25 (1.62) | 4.03 (1.47) | 4.5 (1.64) | <0.001 |
| Education, years | 9.24 (5.19) | 11.6 (5.25) | 13 (4.87) | 11.3 (5.33) | <0.001 |
| APOE ε4 allele^§^ | 56 (28%) | 44 (22%) | 51 (26%) | 151 (25%) | 0.382 |
| BMI (kg/m^2) | 27.5 (4.58) | 27.2 (4.18) | 28 (4.32) | 27.5 (4.36) | 0.237 |
| Diabetes mellitus | 62 (31%) | 63 (32%) | 74 (37%) | 199 (33%) | 0.377 |
| Heart disease | 93 (47%) | 79 (40%) | 88 (44%) | 260 (43%) | 0.383 |
| Hypertension | 171 (86%) | 166 (83%) | 166 (83%) | 503 (84%) | 0.766 |
| Charlson Comorbidity Index | 2.3 (1.52) | 2.13 (1.46) | 2.16 (1.5) | 2.2 (1.49) | 0.466 |
| Incident dementia | 39 (20%) | 25 (13%) | 15 (7.5%) | 79 (13%) | 0.002 |
| P-tau181 level (pg/ml) | 2.5 (1.4) | 2.64 (2.14) | 2.75 (2.52) | 2.63 (2.07) | 0.416 |
| GFAP level (pg/ml) | 183 (100) | 159 (80.3) | 168 (121) | 170 (102) | 0.030 |
| NfL level (pg/ml) | 23 (11.7) | 21.8 (12.5) | 20.7 (12) | 21.8 (12.1) | 0.155 |
| P-tau181/Aβ42 ratio | 0.513 (1.15) | 0.392 (1.11) | 0.417 (1.69) | 0.441 (1.34) | 0.551 |
| Aβ42/40 ratio | 0.0518 (0.0465) | 0.0474 (0.0484) | 0.0493 (0.0277) | 0.0495 (0.0419) | 0.662 |
| PCA1 | 0.142 (1.01) | -0.109 (0.95) | -0.0863 (1) | -0.0249 (0.992) | 0.043 |
| PCA2 | 0.0539 (1.04) | -0.035 (1) | 0.0372 (0.946) | 0.0176 (0.992) | 0.683 |
| Abbreviations (units): O-3, omega-3 (mg/day): O-6, omega-6 (mg/day): PUFA, polyunsaturated fatty acid (mg/day): MUFA, monounsaturated fatty acid (mg/day): APOE ε4, apolipoprotein E epsilon 4 allele: MeDi, Mediterranean diet score: BMI, body mass index (kg/m^2): P-tau181, phosphorylated tau 181 (pg/ml): Aβ42/40, amyloid-beta 42/40 ratio: GFAP, glial fibrillary acidic protein (pg/ml): NfL, neurofilament light chain (pg/ml): PCA1, first principal component (derived from the latter four biomarkers): PCA2, second principal component.  *Missing values were excluded from analysis. Fish intake available for 591 individuals, education available for 598 individuals, APOE ε4 available for 598 individuals, MeDi score available for 591 individuals, BMI available for 522 individuals, Charlson Comorbidity Index available for 597 individuals, P-tau181 available for 598 individuals, Aβ42/40 available for 594 individuals.   †Mean (SD); n (%)  ‡One-way analysis of means (not assuming equal variances); Pearson’s Chi-squared test | | | | | |

**eTable 2.** Summary of participants by baseline O-6 PUFA intake tertile.

|  | **O-6 PUFA Intake Tertile** | | | |  |
| --- | --- | --- | --- | --- | --- |
| **Characteristic**^*^ | **LOW**  N = 200^†^ | **MIDDLE**  N = 199^†^ | **HIGH**  N = 200^†^ | **Overall**  N = 599^†^ | **p-value**^‡^ |
| O-6 PUFA, mg/d | 5380 (2240) | 6040 (2410) | 10500 (4210) | 7310 (3840) | <0.001 |
| Age | 75.7 (6.02) | 73.9 (6) | 71.7 (4.32) | 73.8 (5.74) | <0.001 |
| Women | 146 (73%) | 141 (71%) | 130 (65%) | 417 (70%) | 0.198 |
| Men | 54 (27%) | 58 (29%) | 70 (35%) | 182 (30%) |  |
| Ethnicity |  |  |  |  | <0.001 |
| Non-Hispanic White | 40 (20%) | 57 (29%) | 70 (35%) | 167 (28%) |  |
| Non-Hispanic Black | 23 (12%) | 42 (21%) | 85 (43%) | 150 (25%) |  |
| Hispanic | 137 (69%) | 100 (50%) | 45 (23%) | 282 (47%) |  |
| Energy intake, kcal | 1.43 (0.426) | 1.17 (0.391) | 1.41 (0.474) | 1.34 (0.447) | <0.001 |
| Consumption of 2+ portions of fish, weekly | 81 (41%) | 96 (49%) | 104 (52%) | 281 (48%) | 0.072 |
| O-3 PUFA, mg/d | 200 (209) | 236 (231) | 314 (458) | 250 (323) | 0.005 |
| MUFA, mg/d | 12500 (5750) | 11800 (5320) | 18200 (7010) | 14200 (6710) | <0.001 |
| MeDi score | 4.55 (1.8) | 4.65 (1.71) | 4.31 (1.38) | 4.5 (1.64) | 0.075 |
| Education, years | 9.24 (5.15) | 11.1 (5.43) | 13.5 (4.52) | 11.3 (5.33) | <0.001 |
| APOE ε4 allele^§^ | 50 (25%) | 55 (28%) | 46 (23%) | 151 (25%) | 0.580 |
| BMI (kg/m^2) | 28.2 (4.41) | 26.8 (4.24) | 27.7 (4.35) | 27.5 (4.36) | 0.024 |
| Diabetes mellitus | 59 (30%) | 66 (33%) | 74 (37%) | 199 (33%) | 0.281 |
| Heart disease | 101 (51%) | 83 (42%) | 76 (38%) | 260 (43%) | 0.035 |
| Hypertension | 174 (87%) | 168 (84%) | 161 (81%) | 503 (84%) | 0.204 |
| Charlson Comorbidity Index | 2.43 (1.51) | 2.23 (1.66) | 1.93 (1.24) | 2.2 (1.49) | 0.001 |
| Incident dementia | 39 (20%) | 31 (16%) | 9 (4.5%) | 79 (13%) | <0.001 |
| P-tau181 level (pg/ml) | 2.5 (1.37) | 2.55 (1.91) | 2.83 (2.71) | 2.63 (2.07) | 0.309 |
| GFAP level (pg/ml) | 175 (92.5) | 175 (121) | 160 (89.6) | 170 (102) | 0.215 |
| NfL level (pg/ml) | 22.3 (11) | 22.4 (12.8) | 20.9 (12.4) | 21.8 (12.1) | 0.412 |
| P-tau181/Aβ42 ratio | 0.59 (1.97) | 0.296 (0.227) | 0.436 (1.21) | 0.441 (1.34) | 0.036 |
| Aβ42/40 ratio | 0.0505 (0.0444) | 0.0472 (0.0201) | 0.0508 (0.0537) | 0.0495 (0.0419) | 0.484 |
| PCA1 | 0.0892 (0.984) | -0.0249 (0.975) | -0.124 (1.01) | -0.0249 (0.992) | 0.136 |
| PCA2 | -0.00104 (1.09) | 0.0421 (0.912) | 0.0119 (0.971) | 0.0176 (0.992) | 0.918 |
| Abbreviations (units): O-3, omega-3 (mg/day): O-6, omega-6 (mg/day): PUFA, polyunsaturated fatty acid (mg/day): MUFA, monounsaturated fatty acid (mg/day): APOE ε4, apolipoprotein E epsilon 4 allele: MeDi, Mediterranean diet score: BMI, body mass index (kg/m^2): P-tau181, phosphorylated tau 181 (pg/ml): Aβ42/40, amyloid-beta 42/40 ratio: GFAP, glial fibrillary acidic protein (pg/ml): NfL, neurofilament light chain (pg/ml): PCA1, first principal component (derived from the latter four biomarkers): PCA2, second principal component.  *Missing values were excluded from analysis. Fish intake available for 591 individuals, education available for 598 individuals, APOE ε4 available for 598 individuals, MeDi score available for 591 individuals, BMI available for 522 individuals, Charlson Comorbidity Index available for 597 individuals, P-tau181 available for 598 individuals, Aβ42/40 available for 594 individuals.   †Mean (SD); n (%)  ‡One-way analysis of means (not assuming equal variances); Pearson’s Chi-squared test | | | | | |

**eTable 3.** Baseline biomarker levels by nutrient intake tertiles.

| **Intake** | **Biomarker** | **Model 1^*^** | | **Model 2^†^** | | **Trend^‡^** |
| --- | --- | --- | --- | --- | --- | --- |
|  |  | **Estimate** | **P** | **Estimate** | **P** | **Corrected P** |
| **MUFA intake tertile** | | | | | | |
| HIGH | P-tau181 | 0.028 | 0.291 | 0.025 | 0.358 | 0.688 |
| MIDDLE |  | 0.022 | 0.398 | 0.024 | 0.370 |  |
| HIGH | Aβ42/40 | -0.003 | 0.865 | 0.014 | 0.407 | 0.690 |
| MIDDLE |  | -0.030 | 0.058 | -0.013 | 0.399 |  |
| HIGH | P-tau181/Aβ42 | -0.068 | 0.045 | -0.027 | 0.447 | 0.690 |
| MIDDLE |  | -0.061 | 0.077 | -0.025 | 0.465 |  |
| HIGH | GFAP | -0.045 | 0.045 | -0.027 | 0.224 | 0.628 |
| MIDDLE |  | -0.053 | 0.020 | -0.038 | 0.075 |  |
| HIGH | NfL | -0.051 | 0.017 | -0.023 | 0.283 | 0.659 |
| MIDDLE |  | -0.032 | 0.142 | -0.012 | 0.572 |  |
| HIGH | PCA1 | -0.230 | 0.032 | -0.125 | 0.218 | 0.628 |
| MIDDLE |  | -0.234 | 0.032 | -0.163 | 0.099 |  |
| HIGH | PCA2 | -0.018 | 0.870 | 0.109 | 0.332 | 0.682 |
| MIDDLE |  | -0.080 | 0.467 | 0.030 | 0.780 |  |
| **O-3 PUFA intake tertile** | | | | | | |
| HIGH | P-tau181 | 0.009 | 0.746 | 0.005 | 0.842 | 0.972 |
| MIDDLE |  | -0.033 | 0.214 | -0.038 | 0.138 |  |
| HIGH | Aβ42/40 | -0.036 | 0.026 | -0.002 | 0.906 | 0.972 |
| MIDDLE |  | -0.014 | 0.377 | -0.005 | 0.744 |  |
| HIGH | P-tau181/Aβ42 | -0.029 | 0.405 | 0.027 | 0.449 | 0.690 |
| MIDDLE |  | -0.019 | 0.587 | -0.010 | 0.770 |  |
| HIGH | GFAP | -0.061 | 0.008 | -0.048 | **0.031** | 0.202 |
| MIDDLE |  | -0.043 | 0.058 | -0.044 | **0.035** |  |
| HIGH | NfL | -0.059 | 0.007 | -0.039 | 0.069 | 0.332 |
| MIDDLE |  | -0.052 | 0.017 | -0.053 | **0.008** |  |
| HIGH | PCA1 | -0.302 | 0.005 | -0.139 | 0.170 | 0.628 |
| MIDDLE |  | -0.214 | 0.049 | -0.198 | **0.039** |  |
| HIGH | PCA2 | -0.189 | 0.083 | -0.002 | 0.989 | 0.988 |
| MIDDLE |  | -0.071 | 0.518 | -0.021 | 0.844 |  |
| **O-6 PUFA intake tertile** | | | | | | |
| HIGH | P-tau181 | 0.039 | 0.131 | 0.035 | 0.231 | 0.628 |
| MIDDLE |  | 0.009 | 0.742 | 0.003 | 0.903 |  |
| HIGH | Aβ42/40 | -0.002 | 0.917 | 0.049 | **0.005** | 0.060 |
| MIDDLE |  | 0.002 | 0.901 | 0.033 | **0.040** |  |
| HIGH | P-tau181/Aβ42 | -0.061 | 0.070 | 0.006 | 0.872 | 0.972 |
| MIDDLE |  | -0.056 | 0.107 | -0.020 | 0.571 |  |
| HIGH | GFAP | -0.041 | 0.072 | -0.010 | 0.676 | 0.948 |
| MIDDLE |  | -0.003 | 0.894 | 0.009 | 0.686 |  |
| HIGH | NfL | -0.040 | 0.065 | 0.008 | 0.746 | 0.972 |
| MIDDLE |  | -0.007 | 0.763 | 0.011 | 0.593 |  |
| HIGH | PCA1 | -0.212 | 0.046 | 0.011 | 0.922 | 0.972 |
| MIDDLE |  | -0.079 | 0.478 | 0.015 | 0.880 |  |
| HIGH | PCA2 | 0.014 | 0.894 | 0.333 | **0.005** | 0.060 |
| MIDDLE |  | 0.065 | 0.561 | 0.261 | **0.021** |  |
| Abbreviations (units): O-3, omega-3: O-6, omega-6: PUFA, polyunsaturated fatty acid: MUFA, monounsaturated fatty acid: P-tau181, phosphorylated tau 181 level (pg/ml, log10-transformed): Aβ42/40, amyloid-beta 42/40 ratio (log10-transformed): GFAP, glial fibrillary acidic protein level (pg/ml, log10-transformed): NfL, neurofilament light chain level (pg/ml, log10-transformed): PCA1, first principal component (derived from the latter four biomarkers): PCA2, second principal component.  Generalized linear models were used to evaluate the association of nutrient intake (high and middle compared to low tertiles) with log-transformed baseline biomarker levels. Missing values excluded.  *Model 1 was adjusted for total calorie intake only (N=594 for Aβ42/40, N=599 for GFAP and NfL, N=598 for P-tau181, N=594 for P-tau181/ Aβ42, N=521 for PCAs).  †Model 2 was adjusted for total calorie intake, age, sex, ethnicity, education, and storage time. (N=593 for Aβ42/40, N=598 for GFAP and NfL, N=597 for P-tau181, N=593 for P-tau181/ Aβ42, N=520 for PCAs). ‡A trend analysis of Model 2 was performed, using nutrient tertile as a continuous integer (1-3) (N=427 for Aβ42/40, N=432 for GFAP and NfL, N=430 for P-tau181, N=427 for P-tau181/ Aβ42, N=369 for PCAs). P-values corrected for 21 comparisons using the False Discovery Rate (FDR) are shown. P-values <0.05 are bold. | | | | | | |

**eTable 4**. Longitudinal change in biomarker levels by intake tertiles of subtypes of O-3 PUFAs.

| **Intake** | **Biomarker** | **Model 1^*^** | | **Model 2^†^** | | **Trend^‡^** |
| --- | --- | --- | --- | --- | --- | --- |
|  |  | **Estimate** | **P** | **Estimate** | **P** | **Corrected P** |
| **EPA intake tertile** | | | | | | |
| HIGH | P-tau181 | **-0.032** | **0.005** | **-0.033** | **0.004** | **0.016** |
| MIDDLE |  | -0.013 | 0.309 | -0.013 | 0.318 |  |
| HIGH | Aβ42/40 | 0.014 | 0.054 | 0.013 | 0.067 | 0.141 |
| MIDDLE |  | 0.002 | 0.785 | 0.002 | 0.763 |  |
| HIGH | P-tau181/Aβ42 | **-0.033** | **0.044** | **-0.034** | **0.039** | 0.102 |
| MIDDLE |  | -0.018 | 0.306 | -0.017 | 0.329 |  |
| HIGH | GFAP | -0.011 | 0.259 | -0.012 | 0.235 | 0.413 |
| MIDDLE |  | -0.001 | 0.916 | -0.001 | 0.945 |  |
| HIGH | NfL | -0.007 | 0.510 | -0.007 | 0.486 | 0.602 |
| MIDDLE |  | -0.003 | 0.804 | -0.002 | 0.857 |  |
| HIGH | PCA1 | -0.036 | 0.421 | -0.043 | 0.342 | 0.554 |
| MIDDLE |  | -0.026 | 0.581 | -0.025 | 0.596 |  |
| HIGH | PCA2 | **0.209** | **<0.001** | **0.205** | **<0.001** | **0.002** |
| MIDDLE |  | 0.066 | 0.267 | 0.067 | 0.259 |  |
| **DPA intake tertile** | | | | | | |
| HIGH | P-tau181 | **-0.036** | **0.005** | **-0.037** | **0.004** | **0.016** |
| MIDDLE |  | **-0.027** | **0.039** | **-0.028** | **0.034** |  |
| HIGH | Aβ42/40 | **0.024** | **0.001** | **0.023** | **0.001** | **0.009** |
| MIDDLE |  | 0.012 | 0.130 | 0.012 | 0.131 |  |
| HIGH | P-tau181/Aβ42 | -0.013 | 0.432 | -0.015 | 0.380 | 0.578 |
| MIDDLE |  | -0.015 | 0.407 | -0.016 | 0.375 |  |
| HIGH | GFAP | -0.007 | 0.504 | -0.008 | 0.430 | 0.580 |
| MIDDLE |  | -0.004 | 0.642 | -0.005 | 0.607 |  |
| HIGH | NfL | -0.007 | 0.516 | -0.008 | 0.435 | 0.580 |
| MIDDLE |  | -0.009 | 0.401 | -0.010 | 0.359 |  |
| HIGH | PCA1 | -0.016 | 0.730 | -0.024 | 0.591 | 0.641 |
| MIDDLE |  | -0.033 | 0.488 | -0.037 | 0.438 |  |
| HIGH | PCA2 | **0.230** | **<0.001** | **0.226** | **<0.001** | **0.001** |
| MIDDLE |  | **0.153** | **0.011** | **0.155** | **0.010** |  |
| **DHA intake tertile** | | | | | | |
| HIGH | P-tau181 | **-0.030** | **0.009** | **-0.030** | **0.009** | **0.027** |
| MIDDLE |  | -0.016 | 0.217 | -0.016 | 0.228 |  |
| HIGH | Aβ42/40 | 0.011 | 0.146 | 0.010 | 0.172 | 0.326 |
| MIDDLE |  | 0.006 | 0.361 | 0.007 | 0.337 |  |
| HIGH | P-tau181/Aβ42 | -0.031 | 0.058 | -0.032 | 0.052 | 0.123 |
| MIDDLE |  | -0.023 | 0.184 | -0.022 | 0.203 |  |
| HIGH | GFAP | -0.005 | 0.590 | -0.006 | 0.565 | 0.626 |
| MIDDLE |  | 0.003 | 0.782 | 0.003 | 0.726 |  |
| HIGH | NfL | -0.003 | 0.755 | -0.004 | 0.739 | 0.740 |
| MIDDLE |  | 0.005 | 0.683 | 0.006 | 0.613 |  |
| HIGH | PCA1 | -0.022 | 0.639 | -0.027 | 0.560 | 0.626 |
| MIDDLE |  | -0.006 | 0.904 | -0.002 | 0.961 |  |
| HIGH | PCA2 | **0.173** | **0.002** | **0.169** | **0.003** | **0.015** |
| MIDDLE |  | 0.098 | 0.089 | 0.100 | 0.084 |  |
| Abbreviations (units): EPA, eicosapentaeonic acid: DPA, docosapentaenoic acid: DHA, docosahexaenoic acid: P-tau181, phosphorylated tau 181 level (pg/ml, log10-transformed): Aβ42/40, amyloid-beta 42/40 ratio (log10-transformed): GFAP, glial fibrillary acidic protein level (pg/ml, log10-transformed): NfL, neurofilament light chain level (pg/ml, log10-transformed): PCA1, first principal component (derived from the latter four biomarkers): PCA2, second principal component.component (derived from the latter four biomarkers): PCA2, second principal component. Generalized estimating equations (GEE) were used to evaluate the association of nutrient intake (high and middle compared to low tertiles) with change in log-transformed biomarker levels over time (in visits). Missing values excluded. P-values < 0.05 and their corresponding estimates shown in bold. *Model 1 was adjusted for total energy intake only (N=599 for biomarkers, N = 527 for PCAs). †Model 2 was adjusted for total energy intake, age, sex, ethnicity, education, and storage time (N=598 for biomarkers, N=526 for PCAs).  ‡A trend analysis of Model 2 was performed, using nutrient intake tertile as a continuous integer (1-3). P-values corrected for 21 comparisons using the False Discovery Rate (FDR) are shown. | | | | | | |

**eTable 5.** Longitudinal change of biomarker over time (in years) by nutrient intake (in mg/day).

| **Biomarker** | **Model 1^*^** | | **Model 2^†^** | |
| --- | --- | --- | --- | --- |
|  | **Estimate** | **P** | **Estimate** | **Corrected P** |
| **MUFA intake (mg/day)** | | | | |
| P-tau181 | -\|<0.001\| | 0.597 | -\|<0.001\| | 0.643 |
| Aβ42/40 | **< 0.001** | **0.021** | <0.001 | 0.134 |
| P-tau181/Aβ42 | **0.001** | **0.043** | 0.001 | 0.188 |
| GFAP | -\|<0.001\| | 0.700 | -\|<0.001\| | 0.666 |
| NfL | <0.001 | 0.326 | <0.001 | 0.594 |
| PCA1 | -\|<0.001\| | 0.979 | -\|<0.001\| | 0.823 |
| PCA2 | 0.001 | 0.522 | 0.001 | 0.643 |
| **O-3 PUFA intake (mg/day)** | | | | |
| P-tau181 | **-0.015** | **0.005** | **-0.016** | **0.035** |
| Aβ42/40 | 0.006 | 0.062 | 0.006 | 0.169 |
| P-tau181/Aβ42 | -0.005 | 0.357 | -0.005 | 0.594 |
| GFAP | 0.003 | 0.286 | 0.003 | 0.594 |
| NfL | 0.005 | 0.392 | 0.004 | 0.594 |
| PCA1 | -0.017 | 0.409 | -0.017 | 0.594 |
| PCA2 | **0.069** | **0.005** | **0.071** | **0.035** |
| **O-6 PUFA intake (mg/day)** | | | | |
| P-tau181 | **-0.001** | **0.020** | -0.001 | 0.100 |
| Aβ42/40 | <0.001 | 0.278 | <0.001 | 0.594 |
| P-tau181/Aβ42 | -0.001 | 0.359 | -0.001 | 0.594 |
| GFAP | -\|<0.001\| | 0.516 | -\|<0.001\| | 0.624 |
| NfL | <0.001 | 0.456 | <0.001 | 0.643 |
| PCA1 | -0.003 | 0.081 | -0.003 | 0.207 |
| PCA2 | 0.004 | 0.110 | 0.004 | 0.284 |
| Abbreviations (units): O-3, omega-3 (mg/day): O-6, omega-6 (mg/day): PUFA, polyunsaturated fatty acid (mg/day): MUFA, monounsaturated fatty acid (mg/day): P-tau181, phosphorylated tau 181 level (pg/ml, log10-transformed): Aβ42/40, amyloid-beta 42/40 ratio (log10-transformed): GFAP, glial fibrillary acidic protein level (pg/ml, log10-transformed): NfL, neurofilament light chain level (pg/ml, log10-transformed): PCA1, first principal component (derived from the latter four biomarkers): PCA2, second principal component. Residuals of linear regressions of the fatty acids against total energy intake are shown, in mg/day. Generalized estimating equations (GEE) were used to evaluate the association of nutrient intake with change in log-transformed biomarker levels over time in years. Missing values excluded. P-values < 0.05 and their corresponding estimates shown in bold. *Model 1 was adjusted for total energy intake only (N=599 for biomarkers, N = 527 for PCAs). †Model 2 was adjusted for total energy intake, age, sex, ethnicity, education, and storage time (N=598 for biomarkers, N=526 for PCAs). P-values corrected for 21 comparisons using the False Discovery Rate (FDR) are shown. | | | | |

**eTable 6**. Longitudinal change in biomarker levels by nutrient intake tertile using mixed effects models.

| **Intake** | **Biomarker** | **Model 1^*^** | | **Model 2^†^** | | **Trend^‡^** |  |
| --- | --- | --- | --- | --- | --- | --- | --- |
|  |  | **Estimate** | **P** | **Estimate** | **P** | **Corrected P** |  |
| **MUFA intake tertile** | | | | | | |  |
| HIGH | P-tau181 | -0.015 | 0.196 | -0.016 | 0.181 | 0.394 |  |
| MIDDLE |  | **-0.023** | **0.044** | **-0.024** | **0.040** |  |  |
| HIGH | Aβ42/40 | 0.005 | 0.509 | 0.005 | 0.488 | 0.583 |  |
| MIDDLE |  | 0.008 | 0.273 | 0.007 | 0.315 |  |  |
| HIGH | P-tau181/Aβ42 | 0.026 | 0.128 | 0.026 | 0.125 | 0.305 |  |
| MIDDLE |  | 0.026 | 0.130 | 0.025 | 0.146 |  |  |
| HIGH | GFAP | -0.011 | 0.249 | -0.011 | 0.232 | 0.416 |  |
| MIDDLE |  | -0.011 | 0.225 | -0.012 | 0.210 |  |  |
| HIGH | NfL | 0.016 | 0.125 | 0.016 | 0.131 | 0.305 |  |
| MIDDLE |  | 0.006 | 0.569 | 0.006 | 0.563 |  |  |
| HIGH | PCA1 | 0.031 | 0.494 | 0.031 | 0.493 | 0.583 |  |
| MIDDLE |  | 0.020 | 0.656 | 0.018 | 0.693 |  |  |
| HIGH | PCA2 | 0.009 | 0.865 | 0.010 | 0.855 | 0.856 |  |
| MIDDLE |  | 0.013 | 0.810 | 0.008 | 0.890 |  |  |
| **O-3 intake tertile** | | | | | | |  |
| HIGH | P-tau181 | **-0.037** | **0.001** | **-0.037** | **0.001** | **0.009** |  |
| MIDDLE |  | -0.014 | 0.234 | -0.014 | 0.239 |  |  |
| HIGH | Aβ42/40 | **0.023** | **0.001** | **0.022** | **0.002** | **0.012** |  |
| MIDDLE |  | 0.010 | 0.155 | 0.010 | 0.149 |  |  |
| HIGH | P-tau181/Aβ42 | -0.025 | 0.145 | -0.026 | 0.129 | 0.305 |  |
| MIDDLE |  | -0.014 | 0.394 | -0.014 | 0.420 |  |  |
| HIGH | GFAP | -0.006 | 0.494 | -0.007 | 0.470 | 0.583 |  |
| MIDDLE |  | 0.002 | 0.791 | 0.003 | 0.773 |  |  |
| HIGH | NfL | -0.003 | 0.766 | -0.003 | 0.756 | 0.793 |  |
| MIDDLE |  | 0.008 | 0.464 | 0.008 | 0.460 |  |  |
| HIGH | PCA1 | -0.025 | 0.574 | -0.030 | 0.506 | 0.583 |  |
| MIDDLE |  | 0.002 | 0.963 | 0.004 | 0.932 |  |  |
| HIGH | PCA2 | **0.223** | **0.000** | **0.219** | **0.000** | **0.001** |  |
| MIDDLE |  | **0.117** | **0.033** | **0.118** | **0.030** |  |  |
| **O-6 intake tertile** | | | | | | |  |
| HIGH | P-tau181 | **-0.048** | **0.000** | **-0.049** | **0.000** | **0.001** |  |
| MIDDLE |  | **-0.037** | **0.001** | **-0.037** | **0.001** |  |  |
| HIGH | Aβ42/40 | 0.005 | 0.473 | 0.005 | 0.532 | 0.583 |  |
| MIDDLE |  | 0.000 | 0.964 | 0.000 | 0.974 |  |  |
| HIGH | P-tau181/Aβ42 | -0.012 | 0.498 | -0.013 | 0.460 | 0.583 |  |
| MIDDLE |  | -0.005 | 0.761 | -0.005 | 0.749 |  |  |
| HIGH | GFAP | **-0.024** | **0.010** | **-0.025** | **0.007** | **0.030** |  |
| MIDDLE |  | -0.013 | 0.172 | -0.013 | 0.155 |  |  |
| HIGH | NfL | -0.010 | 0.363 | -0.010 | 0.338 | 0.556 |  |
| MIDDLE |  | -0.015 | 0.154 | -0.016 | 0.144 |  |  |
| HIGH | PCA1 | **-0.088** | **0.049** | **-0.093** | **0.037** | 0.134 |  |
| MIDDLE |  | -0.063 | 0.166 | -0.064 | 0.158 |  |  |
| HIGH | PCA2 | 0.074 | 0.176 | 0.069 | 0.205 | 0.395 |  |
| MIDDLE |  | 0.042 | 0.449 | 0.041 | 0.461 |  |  |
| Abbreviations (units): O-3, omega-3: O-6, omega-6: PUFA, polyunsaturated fatty acid: MUFA, monounsaturated fatty acid: P-tau181, phosphorylated tau 181 level (pg/ml, log10-transformed): Aβ42/40, amyloid-beta 42/40 ratio (log10-transformed): GFAP, glial fibrillary acidic protein level (pg/ml, log10-transformed): NfL, neurofilament light chain level (pg/ml, log10-transformed): PCA1, first principal component (derived from the latter four biomarkers): PCA2, second principal component. Mixed effects models with random effects for repeated measures were used to evaluate the association of nutrient intake (high and middle compared to low tertiles) with change in log-transformed biomarker level over time (in visits). P-values < 0.05 and their corresponding estimates shown in bold. *Model 1 was adjusted for total energy intake only. †Model 2 was adjusted for total energy intake, age, sex, ethnicity, education, and storage time.  ‡A trend analysis of Model 2 was performed, using nutrient intake tertile as a continuous integer (1-3). P-values corrected for 21 comparisons using the False Discovery Rate (FDR) are shown. | | | | | | | |

**eTable 7.** Baseline and longitudinal association between Mediterranean diet score and biomarkers

1. Baseline

|  | **Model 1*** |  | **Model 2**† | | |
| --- | --- | --- | --- | --- | --- |
| **Biomarker** | **Estimate** | **P** | **Estimate** | **P** | **Corrected P**‡ |
| P-tau 181 | -0.005 | 0.403 | -0.002 | 0.772 | 0.869 |
| GFAP | -0.006 | 0.323 | 0.001 | 0.845 | 0.869 |
| NfL | -0.010 | 0.075 | -0.004 | 0.388 | 0.869 |
| Aβ 42/40 | 0.000 | 0.920 | 0.003 | 0.452 | 0.869 |
| P-tau 181 / Aβ42 | -0.005 | 0.558 | 0.001 | 0.869 | 0.869 |
| PCA1 | **-0.053** | **0.046** | -0.010 | 0.688 | 0.869 |
| PCA2 | 0.014 | 0.592 | 0.031 | 0.232 | 0.869 |

1. Longitudinal

|  | **Model 1*** | | **Model 2**† | | |
| --- | --- | --- | --- | --- | --- |
| **Biomarker** | **Estimate** | **P** | **Estimate** | **P** | **Corrected P**‡ |
| P-tau 181 | -0.001 | 0.811 | -0.001 | 0.810 | 0.891 |
| GFAP | 0.001 | 0.733 | 0.001 | 0.714 | 0.891 |
| NfL | -0.002 | 0.334 | -0.002 | 0.328 | 0.581 |
| Aβ 42/40 | 0.001 | 0.323 | 0.001 | 0.328 | 0.581 |
| P-tau 181 / Aβ 42 | -0.004 | 0.325 | -0.004 | 0.332 | 0.581 |
| PCA1 | -0.001 | 0.953 | -0.001 | 0.891 | 0.891 |
| PCA2 | **0.032** | **0.007** | **0.031** | **0.007** | 0.052 |

Abbreviations (units): P-tau181, phosphorylated tau 181 level (pg/ml, log10-transformed): Aβ42/40, amyloid-beta 42/40 ratio (log10-transformed): GFAP, glial fibrillary acidic protein level (pg/ml, log10-transformed): NfL, neurofilament light chain level (pg/ml, log10-transformed): PCA1, first principal component (derived from the latter four biomarkers): PCA2, second principal component.A. Generalized linear models were used to evaluate the association of Mediterranean diet score (0-9) and log-transformed biomarker levels at baseline. P-values < 0.05 and their corresponding estimates shown in bold.

B. Generalized estimating equations were used to evaluate the association of Mediterranean diet score (0-9) at baseline and change in log-transformed biomarker levels over time. P-values < 0.05 and their corresponding estimates shown in bold.
*Model 1 was adjusted for total energy intake only.
†Model 2 was adjusted for total energy intake, age, sex, ethnicity, education, and storage time.
‡ P-values from model 2 corrected for 21 comparisons using the False Discovery Rate (FDR) are shown.

**eTable 8.** Association of baseline nutrient intake with longitudinal biomarker levels, after excluding incident dementia.

| **Intake** | **Biomarker** | **Model 1^*^** | | **Model 2^†^** | | **Trend^‡^** |
| --- | --- | --- | --- | --- | --- | --- |
|  |  | **Estimate** | **P** | **Estimate** | **P** | **Corrected P** |
| **MUFA intake tertile** | | | | | | |
| HIGH | P-tau181 | -0.025 | 0.090 | **-0.030** | **0.042** | 0.148 |
| MIDDLE |  | **-0.034** | **0.011** | **-0.035** | **0.009** |  |
| HIGH | Aβ42/40 | 0.005 | 0.531 | 0.007 | 0.392 | 0.560 |
| MIDDLE |  | 0.011 | 0.184 | 0.013 | 0.127 |  |
| HIGH | P-tau181/Aβ42 | 0.022 | 0.229 | 0.023 | 0.212 | 0.398 |
| MIDDLE |  | 0.020 | 0.315 | 0.015 | 0.429 |  |
| HIGH | GFAP | -0.011 | 0.269 | -0.012 | 0.215 | 0.398 |
| MIDDLE |  | -0.010 | 0.308 | -0.010 | 0.307 |  |
| HIGH | NfL | 0.013 | 0.230 | 0.010 | 0.362 | 0.560 |
| MIDDLE |  | 0.007 | 0.513 | 0.006 | 0.599 |  |
| HIGH | PCA1 | 0.020 | 0.669 | 0.014 | 0.759 | 0.788 |
| MIDDLE |  | 0.005 | 0.920 | 0.004 | 0.940 |  |
| HIGH | PCA2 | 0.018 | 0.763 | 0.030 | 0.617 | 0.779 |
| MIDDLE |  | 0.043 | 0.479 | 0.061 | 0.288 |  |
| **O-3 intake tertile** | | | | | | |
| HIGH | P-tau181 | **-0.043** | **0.001** | **-0.041** | **0.001** | **0.009** |
| MIDDLE |  | -0.016 | 0.290 | -0.013 | 0.396 |  |
| HIGH | Aβ42/40 | **0.023** | **0.006** | **0.019** | **0.021** | 0.068 |
| MIDDLE |  | 0.009 | 0.290 | 0.006 | 0.469 |  |
| HIGH | P-tau181/Aβ42 | -0.023 | 0.184 | -0.020 | 0.227 | 0.398 |
| MIDDLE |  | -0.013 | 0.516 | -0.008 | 0.698 |  |
| HIGH | GFAP | -0.007 | 0.490 | -0.007 | 0.488 | 0.578 |
| MIDDLE |  | 0.004 | 0.677 | 0.006 | 0.568 |  |
| HIGH | NfL | -0.004 | 0.684 | -0.003 | 0.761 | 0.788 |
| MIDDLE |  | 0.007 | 0.567 | 0.009 | 0.463 |  |
| HIGH | PCA1 | -0.033 | 0.479 | -0.036 | 0.447 | 0.560 |
| MIDDLE |  | 0.007 | 0.892 | 0.014 | 0.780 |  |
| HIGH | PCA2 | **0.212** | **0.000** | **0.178** | **0.002** | **0.010** |
| MIDDLE |  | 0.097 | 0.137 | 0.068 | 0.270 |  |
| **O-6 intake tertile** | | | | | | |
| HIGH | P-tau181 | **-0.059** | **0.000** | **-0.061** | **0.000** | **0.001** |
| MIDDLE |  | **-0.048** | **0.001** | **-0.052** | **0.000** |  |
| HIGH | Aβ42/40 | 0.005 | 0.569 | 0.002 | 0.838 | 0.842 |
| MIDDLE |  | 0.002 | 0.796 | 0.001 | 0.858 |  |
| HIGH | P-tau181/Aβ42 | -0.027 | 0.159 | -0.023 | 0.216 | 0.398 |
| MIDDLE |  | -0.015 | 0.431 | -0.010 | 0.588 |  |
| HIGH | GFAP | **-0.026** | **0.006** | **-0.028** | **0.003** | **0.020** |
| MIDDLE |  | -0.017 | 0.092 | -0.019 | 0.065 |  |
| HIGH | NfL | -0.015 | 0.152 | -0.016 | 0.129 | 0.383 |
| MIDDLE |  | -0.015 | 0.182 | -0.018 | 0.105 |  |
| HIGH | PCA1 | **-0.121** | **0.007** | **-0.130** | **0.004** | **0.022** |
| MIDDLE |  | -0.091 | 0.060 | **-0.101** | **0.035** |  |
| HIGH | PCA2 | 0.082 | 0.181 | 0.050 | 0.375 | 0.560 |
| MIDDLE |  | 0.059 | 0.343 | 0.040 | 0.473 |  |
| Abbreviations (units): O-3, omega-3: O-6, omega-6: PUFA, polyunsaturated fatty acid: MUFA, monounsaturated fatty acid: P-tau181, phosphorylated tau 181 level (pg/ml, log10-transformed): Aβ42/40, amyloid-beta 42/40 ratio (log10-transformed): GFAP, glial fibrillary acidic protein level (pg/ml, log10-transformed): NfL, neurofilament light chain level (pg/ml, log10-transformed): PCA1, first principal component (derived from the latter four biomarkers): PCA2, second principal component. Generalized estimating equations (GEE) were used to evaluate the association of nutrient intake (high and middle compared to low tertiles) with change in biomarker level over time (in visits), after excluding individuals who developed dementia during follow up visits. Missing values excluded. P-values < 0.05 shown in bold.  *Model 1 was adjusted for total energy intake only (N=520 for biomarkers, N = 482 for PCAs). †Model 2 was adjusted for total energy intake, age, sex, ethnicity, education, and storage time (N=513 for biomarkers, N=475 for PCAs).  ‡A trend analysis of Model 2 was performed, using nutrient intake tertile as a continuous integer (1-3). P-values corrected for 21 comparisons using the False Discovery Rate (FDR) are shown. | | | | | | |

**eTable 9.** Longitudinal change in biomarkers by baseline nutrient intake tertile, additionally adjusted for the Charlson Comorbidity Index.

| **Intake** | **Biomarker** | **Estimate** | **P** | **Trend analysis, corrected P*** |
| --- | --- | --- | --- | --- |
| **MUFA intake tertile** | | | | |
| HIGH | P-tau181 | -0.019 | 0.158 | 0.321 |
| MIDDLE |  | **-0.026** | **0.025** |  |
| HIGH | Aβ42/40 | 0.006 | 0.382 | 0.568 |
| MIDDLE |  | 0.008 | 0.275 |  |
| HIGH | P-tau181/Aβ42 | 0.026 | 0.133 | 0.314 |
| MIDDLE |  | 0.025 | 0.159 |  |
| HIGH | GFAP | -0.014 | 0.126 | 0.314 |
| MIDDLE |  | -0.013 | 0.159 |  |
| HIGH | NfL | 0.014 | 0.184 | 0.321 |
| MIDDLE |  | 0.006 | 0.567 |  |
| HIGH | PCA1 | 0.022 | 0.617 | 0.687 |
| MIDDLE |  | 0.016 | 0.728 |  |
| HIGH | PCA2 | 0.017 | 0.764 | 0.798 |
| MIDDLE |  | 0.016 | 0.776 |  |
| **O-3 intake tertile** | | | | |
| HIGH | P-tau181 | **-0.038** | **0.001** | **0.006** |
| MIDDLE |  | -0.014 | 0.312 |  |
| HIGH | Aβ42/40 | **0.022** | **0.003** | **0.013** |
| MIDDLE |  | 0.010 | 0.216 |  |
| HIGH | P-tau181/Aβ42 | -0.026 | 0.122 | 0.314 |
| MIDDLE |  | -0.014 | 0.438 |  |
| HIGH | GFAP | -0.006 | 0.542 | 0.635 |
| MIDDLE |  | 0.004 | 0.658 |  |
| HIGH | NfL | -0.003 | 0.798 | 0.798 |
| MIDDLE |  | 0.009 | 0.415 |  |
| HIGH | PCA1 | -0.027 | 0.553 | 0.635 |
| MIDDLE |  | 0.009 | 0.843 |  |
| HIGH | PCA2 | **0.221** | **0.000** | **0.001** |
| MIDDLE |  | 0.113 | 0.062 |  |
| **O-6 intake tertile** | | | | |
| HIGH | P-tau181 | **-0.050** | **0.000** | **0.001** |
| MIDDLE |  | **-0.037** | **0.003** |  |
| HIGH | Aβ42/40 | 0.006 | 0.449 | 0.625 |
| MIDDLE |  | 0.001 | 0.888 |  |
| HIGH | P-tau181/Aβ42 | -0.012 | 0.509 | 0.635 |
| MIDDLE |  | -0.005 | 0.753 |  |
| HIGH | GFAP | **-0.027** | **0.002** | **0.013** |
| MIDDLE |  | -0.015 | 0.128 |  |
| HIGH | NfL | -0.011 | 0.286 | 0.468 |
| MIDDLE |  | -0.015 | 0.159 |  |
| HIGH | PCA1 | **-0.095** | **0.028** | 0.102 |
| MIDDLE |  | -0.064 | 0.170 |  |
| HIGH | PCA2 | 0.077 | 0.181 | 0.321 |
| MIDDLE |  | 0.046 | 0.413 |  |

Abbreviations (units): O-3, omega-3: O-6, omega-6: PUFA, polyunsaturated fatty acid: MUFA, monounsaturated fatty acid: P-tau181, phosphorylated tau 181 level (pg/ml, log10-transformed): Aβ42/40, amyloid-beta 42/40 ratio (log10-transformed): GFAP, glial fibrillary acidic protein level (pg/ml, log10-transformed): NfL, neurofilament light chain level (pg/ml, log10-transformed): PCA1, first principal component (derived from the latter four biomarkers): PCA2, second principal component.
Generalized estimating equations (GEE) were used to evaluate the association of nutrient intake (high and middle compared to low tertiles) with change in biomarker level over time (in visits). Missing values excluded. P-values < 0.05 and their associated estimates are in bold.
The model was adjusted for total energy intake, age, sex, ethnicity, education, storage time, and the Charlson Comorbidity Index (N=596 for biomarkers, N=524 for PCAs).
*A trend analysis of the model was performed, using nutrient intake tertile as a continuous integer (1-3). P-values corrected for 21 comparisons using the False Discovery Rate (FDR) are shown.

**eTable 10.** Interaction of nutrient intake tertile and sex on biomarker levels at baseline.

| **Intake** | **Biomarker** | **Model 1^*^** | | **Model 2^†^** | | **Trend^‡^** |
| --- | --- | --- | --- | --- | --- | --- |
|  |  | **Estimate** | **P** | **Estimate** | **P** | **Corrected P** |
| **MUFA intake tertile** | | | | | | |
| HIGH | P-tau181 | -0.108 | 0.056 | -0.082 | 0.140 | 0.505 |
| MIDDLE |  | -0.045 | 0.430 | -0.004 | 0.948 |  |
| HIGH | Aβ42/40 | -0.020 | 0.562 | -0.050 | 0.136 | 0.505 |
| MIDDLE |  | -0.011 | 0.763 | -0.042 | 0.210 |  |
| HIGH | P-tau181/Aβ42 | 0.018 | 0.812 | 0.006 | 0.937 | 0.950 |
| MIDDLE |  | -0.007 | 0.930 | -0.004 | 0.960 |  |
| HIGH | GFAP | -0.006 | 0.892 | 0.009 | 0.845 | 0.950 |
| MIDDLE |  | -0.022 | 0.652 | 0.010 | 0.825 |  |
| HIGH | NfL | 0.011 | 0.812 | 0.032 | 0.457 | 0.673 |
| MIDDLE |  | -0.038 | 0.420 | -0.007 | 0.878 |  |
| HIGH | PCA1 | 0.015 | 0.949 | 0.075 | 0.716 | 0.880 |
| MIDDLE |  | -0.151 | 0.513 | -0.037 | 0.860 |  |
| HIGH | PCA2 | 0.021 | 0.928 | -0.099 | 0.663 | 0.850 |
| MIDDLE |  | 0.115 | 0.628 | -0.024 | 0.917 |  |
| **O-3 PUFA intake tertile** | | | | | | |
| HIGH | P-tau181 | -0.005 | 0.928 | 0.005 | 0.925 | 0.950 |
| MIDDLE |  | -0.003 | 0.963 | 0.006 | 0.909 |  |
| HIGH | Aβ42/40 | -0.059 | 0.090 | -0.064 | 0.054 | 0.505 |
| MIDDLE |  | -0.024 | 0.485 | -0.045 | 0.175 |  |
| HIGH | P-tau181/Aβ42 | -0.110 | 0.139 | -0.113 | 0.121 | 0.505 |
| MIDDLE |  | -0.065 | 0.381 | -0.086 | 0.233 |  |
| HIGH | GFAP | 0.030 | 0.532 | 0.038 | 0.396 | 0.673 |
| MIDDLE |  | -0.067 | 0.154 | -0.070 | 0.119 |  |
| HIGH | NfL | -0.047 | 0.310 | -0.036 | 0.410 | 0.673 |
| MIDDLE |  | -0.065 | 0.161 | -0.061 | 0.156 |  |
| HIGH | PCA1 | -0.383 | 0.090 | -0.382 | 0.060 | 0.505 |
| MIDDLE |  | **-0.468** | **0.039** | **-0.535** | **0.009** |  |
| HIGH | PCA2 | -0.249 | 0.283 | -0.279 | 0.218 | 0.545 |
| MIDDLE |  | 0.112 | 0.630 | -0.013 | 0.955 |  |
| **O-6 PUFA intake tertile** | | | | | | |
| HIGH | P-tau181 | -0.054 | 0.346 | -0.045 | 0.417 | 0.673 |
| MIDDLE |  | -0.005 | 0.926 | 0.020 | 0.717 |  |
| HIGH | Aβ42/40 | -0.012 | 0.733 | -0.024 | 0.459 | 0.673 |
| MIDDLE |  | 0.015 | 0.664 | -0.006 | 0.850 |  |
| HIGH | P-tau181/Aβ42 | -0.087 | 0.237 | -0.098 | 0.177 | 0.505 |
| MIDDLE |  | 0.041 | 0.589 | 0.041 | 0.579 |  |
| HIGH | GFAP | 0.020 | 0.681 | 0.031 | 0.494 | 0.673 |
| MIDDLE |  | 0.007 | 0.883 | 0.024 | 0.596 |  |
| HIGH | NfL | 0.046 | 0.329 | 0.052 | 0.228 | 0.545 |
| MIDDLE |  | -0.029 | 0.544 | -0.005 | 0.910 |  |
| HIGH | PCA1 | 0.100 | 0.657 | 0.063 | 0.756 | 0.880 |
| MIDDLE |  | -0.020 | 0.931 | 0.045 | 0.830 |  |
| HIGH | PCA2 | 0.182 | 0.432 | 0.154 | 0.489 | 0.673 |
| MIDDLE |  | 0.175 | 0.466 | 0.092 | 0.692 |  |

Abbreviations (units): O-3, omega-3: O-6, omega-6: PUFA, polyunsaturated fatty acid: MUFA, monounsaturated fatty acid: P-tau181, phosphorylated tau 181 level (pg/ml, log10-transformed): Aβ42/40, amyloid-beta 42/40 ratio (log10-transformed): GFAP, glial fibrillary acidic protein level (pg/ml, log10-transformed): NfL, neurofilament light chain level (pg/ml, log10-transformed): PCA1, first principal component (derived from the latter four biomarkers): PCA2, second principal component.
Generalized linear models were used to evaluate the association of the interaction of sex and nutrient intake (high and middle compared to low tertiles) on log-transformed biomarker levels at baseline. Missing values excluded. P-values <0.05 and their corresponding estimates are bold.

*Model 1 was adjusted for total calorie intake only (N=594 for Aβ42/40, N=599 for GFAP and NfL, N=598 for P-tau181, N=594 for P-tau181/ Aβ42, N=521 for PCAs).

†Model 2 was adjusted for total calorie intake, age, sex, ethnicity, education, and storage time. (N=593 for Ab42/40, N=598 for GFAP and NfL, N=597 for P-tau181, N=593 for P-tau181/ Ab42, N=520 for PCAs).
‡A trend analysis of Model 2 was performed, using nutrient tertile as a continuous integer (1-3). P-values corrected for 21 comparisons using the False Discovery Rate (FDR) are shown.
